# Oxygen-Reconstituted Active Species of Single-Atom Cu Catalysts for Oxygen Reduction Reaction

**DOI:** 10.34133/2020/7593023

**Published:** 2020-10-05

**Authors:** Liu Yang, Haoxiang Xu, Huibing Liu, Xiaofei Zeng, Daojian Cheng, Yan Huang, Lirong Zheng, Rui Cao, Dapeng Cao

**Affiliations:** ^1^Beijing Advanced Innovation Center for Soft Matter Science and Engineering, State Key Laboratory of Organic-Inorganic Composites, Beijing University of Chemical Technology, Beijing 100029, China; ^2^Beijing Synchrotron Radiation Facility, Institute of High Energy Physics, Chinese Academy of Sciences, Beijing 100049, China; ^3^Stanford Synchrotron Radiation Lightsource, SLAC National Accelerator Laboratory, Menlo Park, California 94025, USA

## Abstract

Identification of an active center of catalysts under realistic working conditions of oxygen reduction reaction (ORR) still remains a great challenge and unclear. Herein, we synthesize the Cu single atom embedded on nitrogen-doped graphene-like matrix electrocatalyst (abbreviated as SA-Cu/NG). The results show that SA-Cu/NG possesses a higher ORR capability than 20% Pt/C at alkaline solution while the inferior activity to 20% Pt/C at acidic medium. Based on the experiment and simulation calculation, we identify the atomic structure of Cu-N_2_C_2_ in SA-Cu/NG and for the first time unravels that the oxygen-reconstituted Cu-N_2_C_2_-O structure is really the active species of alkaline ORR, while the oxygen reconstitution does not happen at acidic medium. The finding of oxygen-reconstituted active species of SA-Cu/NG at alkaline media successfully unveils the bottleneck puzzle of why the performance of ORR catalysts at alkaline solution is better than that at acidic media, which provides new physical insight into the development of new ORR catalysts.

## 1. Introduction

When energy consumption is rapidly rising, the resulting environmental issues have become serious [1]. Therefore, developing green and sustainable energies is the key to solve existing questions [2]. Oxygen reduction reaction (ORR) is considered as a crucial electrochemical reaction, and it often determines the performance of new energy devices like fuel cells and other energy storage equipment [3–7]. At present, the platinum (Pt) relative nanomaterials are still the state-of-the-art catalysts for the ORR, which significantly hinders the commercialization of these devices due to the rareness of precious Pt [8].

Recently, atomically dispersed transition metal/nitrogen- (N-) doped porous carbons (M-N-C) are regarded as valuable nonnoble metal catalysts to substitute for commercial Pt [9–12], including Fe- [13–18], Co- [19–21], Mn- [22, 23], and Ru- [24, 25] based M-N-C catalysts. Inspired that Cu compounds can effectively catalyze the reductive activation of O_2_ in enzymes and the protein lactase, increasing investigations indicate that the single-atom Cu catalysts (Cu-SACs) can also serve as excellent ORR catalysts. For example, Cu-N-C moieties can become highly efficient active sites by adjusting the valance state of Cu atom toward Cu(II) or Cu(I) by engineering coordination environment of Cu-N-C moieties [26–28]. And some useful strategies have also been developed to synthesize the Cu-SACs trapped on nitrogen-rich porous carbon with large-scale or high load contents [29–31]. Although these studies have shown that the Cu-SACs possess high ORR activity, the critical question of “what are the realistic active sites of Cu-SACs for ORR?” is unclear yet and in dispute. Due to the high similarities of bond lengths of metal-nitrogen/metal-carbon/metal-oxygen, as well as pyridine/pyrrole species, the extended X-ray absorption fine structure (EXAFS) fitting still is not able to accurately analyze the precise configuration of these samples in R space. Moreover, the atomically dispersed M-N-C catalysts from different synthesis methods also often possess different coordination environments and active species. In fact, the experimental explanation on active species of Cu-SACs for ORR is still severely dependent on X-ray absorption near-edge structure (XANES) and EXAFS without better techniques. Among them, the carbon-hosted CuN_4_ moiety has been proposed to be considered as an active center of Cu-SACs [27–29, 31, 32], based on the coordination number (4) of Cu in the microstructure. Nevertheless, with a computational hydrogen electrode (CHE) model, the well-established Cu-SAC model with CuN_4_ center possesses rather inferior ORR activity to Pt(111) according to the theoretical onset potential (0.25~0.43 V vs. 0.79 V) [27, 28, 31–33], which indicates that the atomic-level understanding on the active center of Cu-SACs boosting ORR remains controversial. More importantly, previous studies demonstrated that numerous catalysts experienced reconstruction during the test operation, probably originating from some physical and chemical aspects, such as pH, potential polarization, and adsorbates [34–36]. Therefore, identification of the definite active-site structure and thermodynamics intermediate transformation on Cu-N-C catalysts under ORR working conditions are required urgently, which is significantly important for intelligent fabrication of fresh ORR SACs.

Herein, we use the surfactant-assisted synthesis method to successfully prepare the single-atom Cu embedded on an N-doped graphene-like matrix (abbreviated as SA-Cu/NG). The synthesized catalyst displays superior ORR property than 20% Pt/C at 0.1 M KOH electrolyte, while inferior ORR performance to 20% Pt/C at acidic solution. Based on the XAS and the systematic search for possible configurations by density functional theory (DFT) calculations, we identified the atomic structure of the CuN_2_C_2_ moiety embedded in porous graphene-like nanosheets for SA-Cu/NG. We also found that the reconstitution of active species of Cu-N_2_C_2_ under ORR working condition was induced by dynamic adsorption of atomic oxygen (O^∗^) intermediate on the Cu-C site (marked as Cu-N_2_C_2_-O), and the oxygen-reconstituted Cu-N_2_C_2_-O structure is the real active species of SA-Cu/NG for ORR at alkaline condition. The oxygen-reconstituted Cu-N_2_C_2_-O structure accounts for superior ORR activity of SA-Cu/NG to Pt/C at alkaline condition, while the virgin Cu-N_2_C_2_ is responsible for inferior activity to Pt/C at acidic medium. The finding of oxygen-reconstituted active species at alkaline media unveils the bottleneck puzzle of why the activity of the ORR catalyst at alkaline condition is always superior to the one at acidic solution.

## 2. Results and Discussion

### 2.1. The Characterization of SA-Cu/NG

**Figure 1:**
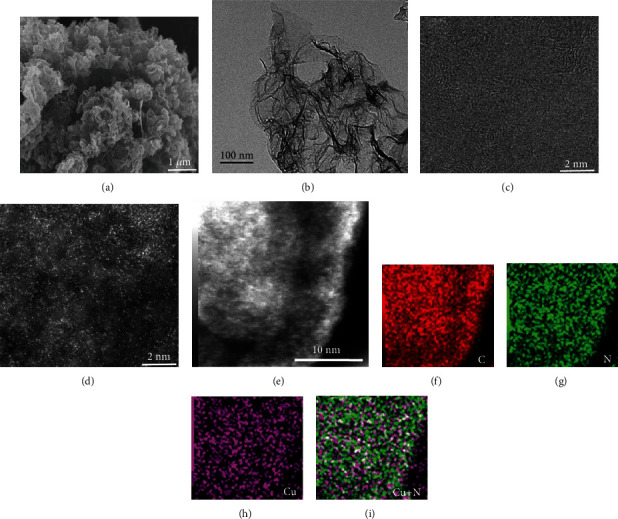
Morphologies of samples: (a) the SEM image, (b) TEM image, (c) HRTEM image, and (d) HAADF STEM image for SA-Cu/NG. (e–i) EELS mapping of C, N, Cu, and overlying Cu and N for SA-Cu/NG sample.

The single-atom Cu embedded on N-doped graphene-like matrix (SA-Cu/NG) was synthesized via the modified surfactant-assisted method from our group, and the detailed synthesis steps are presented in Supplementary Materials (available here). The scanning electron microscope (SEM) image displays that the SA-Cu/NG possesses petal-like morphologies that are made up of porous thin nanosheets (Figure 1(a)). The transmission electron microscope (TEM) image exhibits the graphene-like flakes of the as-prepared SA-Cu/NG sample without aggregation of Cu nanoparticles (Figure 1(b)), even under high-resolution TEM (HRTEM) in Figure 1(c). This phenomenon is also proved by XRD, where no obvious diffraction peaks are detected for SA-Cu/NG sample in Figure S1. Impressively, numerous atomic-level brightened dots are detected at a high-angle annular dark-field (HAADF) pattern (Figure 1(d)), corresponding to heavy copper atoms (Figure 1(d) and Figure S2). Furthermore, the EELS mappings (Figures 1(e)–1(i)) reveal that the Cu, C, and N are homogeneously dispersed on the surface of SA-Cu/NG, and the overlay mode image (Figure 1(i)) discloses that the Cu atoms are surrounded with N.

To explore the valence state, composition of the element sample, and surrounding coordination environment of Cu, X-ray photoelectron spectroscopy (XPS), XANES, and EXAFS are applied. In Figure S3 and Table S1, the XPS spectrum displays that the SA-Cu/NG contains nitrogen of 6.34 at% and Cu of 0.56 at%. The N 1s spectrum for SA-Cu/NG can be fitted to four types [37]. Notably, after atomic Cu is doped into porous carbon, the proportion of pyrrolic N/Cu-N increased, indicating that the Cu-N moiety is formed in the SA-Cu/NG sample (Figures S4 and S5). Moreover, the Cu 2p curve shows two peaks at 935.4 eV and 955.2 eV, attributed to Cu^2+2^p_3/2_ and 2p_1/2_. Cu 2p satellites at 944.3 eV indicate the existence of an unfilled electron (Figure S6) [26, 38].

**Figure 2:**
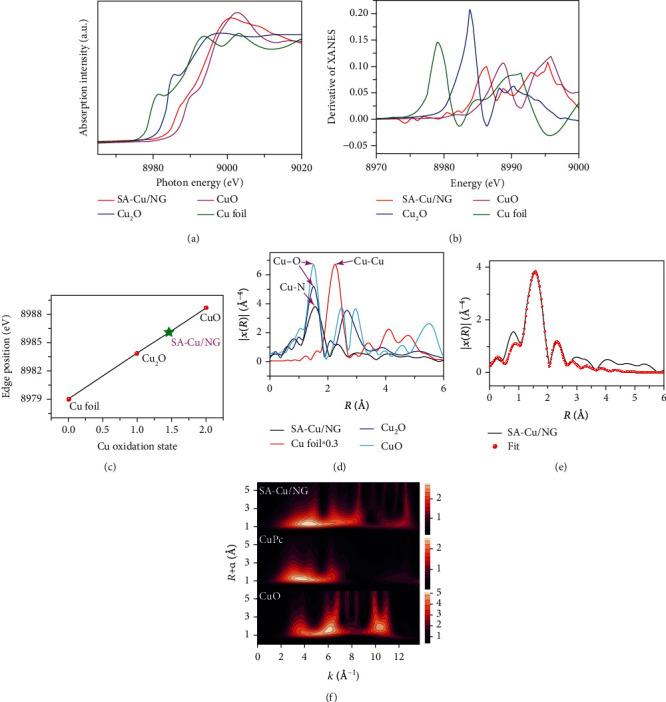
The structure characterization of samples: (a) XANES spectra, (b) derivative of normalized XANES of Cu K-edge, (c) the fitting valence of Cu extracted from XANES, (d) Fourier transforms of Cu K-edge spectra, (e) wavelet transform (WT), and (f) EXAFS fitting curves of SA-Cu/NG and standard samples of CuPc and CuO.

The Cu K-edge XANES and the EXAFS of SA-Cu/NG are also used to explore the local structure of SA-Cu/NG with the standard Cu foil, Cu_2_O, and CuO as counterparts. The valence of Cu in SA-Cu/NG is found between Cu(I) and Cu(II), because the XANES curve for SA-Cu/NG shifts to the higher energy than the Cu foil and Cu_2_O, while slightly lower energy than standard CuO (Figure 2(a)). To calculate the Cu oxidation valence, the fitting curves derived from the first maximum in the first-order derivative of Cu K-edge XANES are depicted in Figures 2(b) and 2(c), and the Cu average valence state of SA-Cu/NG is about +1.46. The intuitionistic structural information on Cu atoms can be obtained from the EXAFS via Fourier transforms (FT) (Figure 2(d)), where the SA-Cu/NG exhibits a peak around 1.56 Å (phase uncorrected) attributed to the Cu-N bond (or Cu-C bond), and a satellite peak corresponds to second shell Cu-C bond (at about 2.35 Å). No obvious peak around 2.2 Å (Cu-Cu bond) is observed in the SA-Cu/NG sample compared to what is found in the Cu_2_O and CuO (Figure 2(d), blue and cyan trances), which suggests that the Cu atoms do not aggregate into nanoparticles that are connected by Cu-Cu bond. The observation is in agreement with the XRD and STEM. EXAFS fitting on the first shell of SA-Cu/NG catalyst (Figure 2(e)) exhibits that the configuration for the SA-Cu/NG catalysts is one Cu coordinated with four surrounding nitrogen or carbon, and the fitting information is gathered in Table S2. EXAFS wavelet transform (WT) analysis is used as a precise technique to distinguish the similar backscattering atoms based on resolutions in both *k* and *R* spaces. As shown in Figure 2(f), the Cu-SA/NG exhibits one obvious maximum at approximatively 4.5 Å^−1^, which is ascribed to the Cu-N(C) bonding, and no Cu-Cu bond is discovered. Impressively, compared with the CuPc standard sample, SA-Cu/NG shows a slightly shift position in maximum intensity, indicating that atomic Cu in the SA-Cu/NG sample coordinates not only with nitrogen but also with the adjacent carbon. However, this phenomenon cannot be analyzed from *R* space of Cu EXAFS experimentally.

As well known, the performance of the catalysts is related to the intrinsic activity and the number of active centers accessible, so the porosity of the samples is also explored. Interestingly, the SA-Cu/NG has a high surface area (884 m^2^ g^−1^) and excellent pore property (1.97 cm^3^ g^−1^) (Figures S7 and S8 and Table S3). This high porosity of SA-Cu/NG is beneficial to contact abundant catalytic centers and transfer electrons [39].

### 2.2. Alkaline ORR Tests

**Figure 3:**
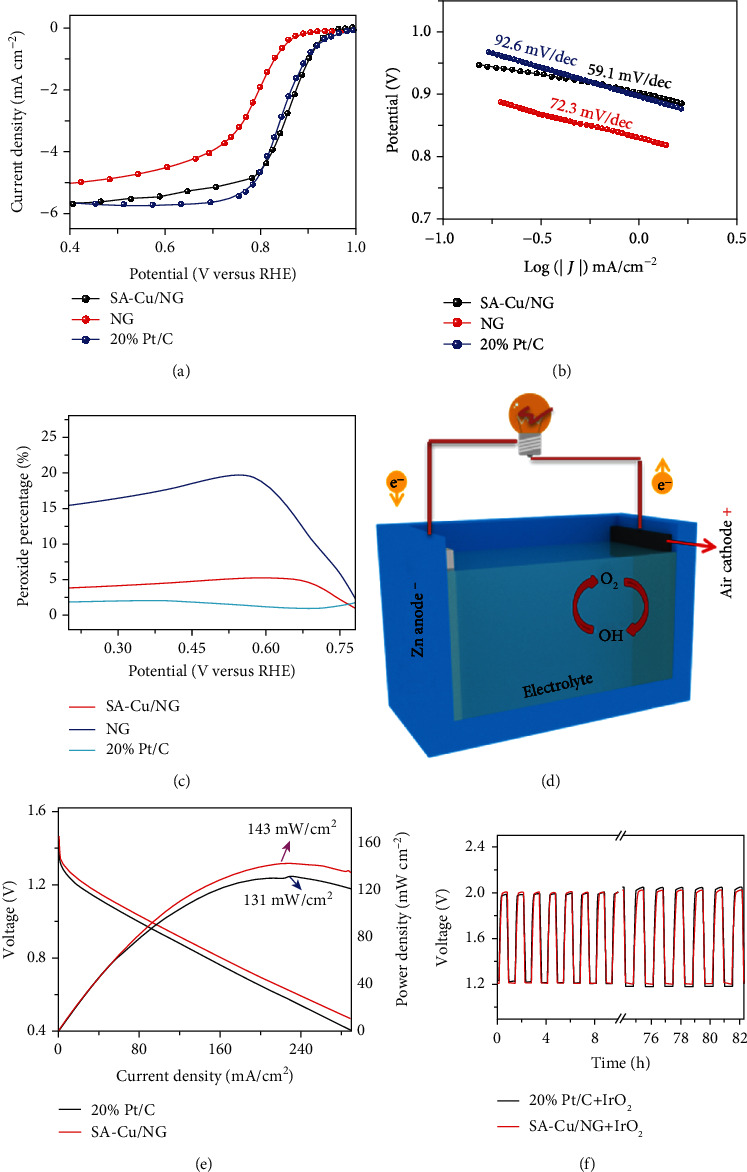
Electrocatalytic performance of samples and related devices. (a) LSV and (b) Tafel slope of fabricated samples and 20% Pt/C in oxygen-saturated 0.1 M KOH electrolyte with 1600 rpm. (c) The HO_2_^−^ yields of fabricated samples and 20% Pt/C derived from RRDE. (d) The schematic diagram of Zn-air battery. (e) The discharging plots of SA-Cu/NG-based battery as well as 20% Pt/C constructed one. (f) The total 82 h voltage curves as well as the enlarged 1-10 h and 75-82 h cycle voltage for SA-Cu/NG+IrO_2_ and contrast batteries.

The electrochemical tests are conducted in 0.1 M KOH solution to investigate the ORR performance of SA-Cu/NG, NG, and 20% Pt/C. In Figure 3(a), the NG displays the general electrochemical curve, verified by the half-wave potential (*E*_1/2_) of 0.8 V (versus RHE), indicating that the N-doped carbon can catalyze ORR but not highly efficient. Impressively, when the small quantity of Cu is introduced into the NG sample, a leap increase of ORR activity is observed for the SA-Cu/NG sample, which indicates that the SA-Cu/NG with mixed valence is highly efficient for ORR. *E*_1/2_ of 0.856 V (versus RHE) is obtained for SA-Cu/NG, more excellent than 20% Pt (*E*_1/2_ of 0.844 V, versus RHE). The excellent activity for SA-Cu/NG is comparable with the majority previously published for nonnoble metal electrocatalysts [10, 40–48]. Remarkably, the SA-Cu/NG also shows large kinetic current density (*J*_k_) of 6.84 mA/cm^2^ at 0.85 V (versus RHE), which is 11.4 times of NG (0.6 mA/cm^2^) and 1.4-folds of 20% Pt/C (5 mA/cm^2^), respectively (Figure S9). A small Tafel slope (59.1 mV dec^−1^) further verifies the more outstanding activity of SA-Cu/NG to 20% Pt/C (92.6 mV decade^−1^) (Figure 3(b)). The selectivity and kinetic of SA-Cu/NG are studied. As shown in Figures S10 and S11, based on slopes of the Koutecky-Levich (K-L) equation, the transfer electron number (*n*) is obtained. The SA-Cu/NG is 3.95 at 0.7 V (versus RHE), better than NG (3.74 at 0.3 V, versus RHE) and comparable to commercial Pt/C catalyst. This phenomenon indicates the outstanding 4e^−^ selectivity of SA-Cu/NG in 0.1 M KOH. Moreover, Figure 3(c) shows that the RRDE-measured HO_2_^−^ yields for SA-Cu/NG are not more than 5.3% within 0.2-0.8 V in 0.1 M KOH, which is extremely lower than the NG sample (<19%). Similarly, *n* for SA-Cu/NG from RRDE measurement is 3.89-3.96 in the scope of the test (Figure S12), in agreement with the RDE results. The electron transfer number and HO_2_^−^ yields demonstrate that SA-Cu/NG possesses a 4e^−^ reaction path for alkaline ORR.

Apart from the ORR performance, the long-term durability and methanol resistance ability of the catalysts are another pivotal concern for the actual applications for corresponding devices. As expected, the retention of current density of SA-Cu/NG electrodes reaches nearly 92% after the continuous 40000 s testing, apparently more stable than 20% Pt/C of 81.8% (Figure S13). Moreover, the chronoamperometric curves of 20% Pt/C drop rapidly when methanol is added at 400 s, while the SA-Cu/NG has no effect, indicating that the SA-Cu/NG possesses robust immunity toward methanol crossover (Figure S14).

### 2.3. Zn-Air Battery

We also assembled the primary Zn-air battery to deeply assess the catalytic activity of the SA-Cu/NG catalyst (Figure 3(d)). As illustrated in Figure S15, the open circuit voltage of the SA-Cu/NG-based battery attains 1.46 V and better discharge performance than the 20% Pt/C counterpart, with a 31 mV and 35 mV positive voltage at 50 mA/cm^2^ and 100 mA/cm^2^ compared to that of the 20% Pt/C counterpart (Figure 3(e)). Moreover, the peak power density reaches 143 mW/cm^2^ at 221 mA/cm^2^, which is 12 mW/cm^2^ larger than the 20% Pt/C assembled battery (Figure 3(e)). To explore the cycle stability, a recyclable Zn-air battery is also assembled by using the combination of SA-Cu/NG and IrO_2_ as air cathode, and it exhibits robust cycling stability for continuous working for 90 h under 5 mA/cm^2^ (Figure S16). Specifically, the round-trip overpotential of SA-Cu/NG+IrO_2_-based battery increases to 0.82 V at about 80 h from the initial 0.78 V. In contrast, a 15.5% increase of overpotential is observed for the 20% Pt/C+IrO_2_-based system (Figure 3(f)). Impressively, as shown in Figure S17, three SA-Cu/NG-based batteries in series can light up one LED display screen (the rated voltage 3.7 V). Actually, the SA-Cu/NG catalyst exhibits outstanding ORR performance while also possesses robust durability in the Zn-air battery, which shows a promising perspective in the application of practical devices.

### 2.4. Identification of Active Center of SA-Cu/NG by DFT Calculation and Experiments

The above results indicate that SA-Cu/NG possesses outstanding ORR performance. Notably, it is not sensitive enough to obtain accurate coordination environment of Cu single atom by fitting *R* space of samples with high similarity of the lengths of Cu-C, Cu-N, and Cu-O bonds, as well as pyridine/pyrrole species. Therefore, we used DFT calculations to identify the atomically precise configuration of the active species of the SA-Cu/NG catalyst. In view of the EXAFS fitting on the nearest shell coordination of Cu atom, the coordination number (CN) of Cu atom is around four, possibly including Cu-N/Cu-C/Cu-O bonds. To precisely identify the active species, we constructed 32 types of all possible structures of Cu‐N_*x*_C_*y*_O_z_, as shown in Figure S18, in which one Cu atom of the SA-Cu/NG is connected to four nearest-neighbor atoms. All the structures are optimized using DFT relaxation. We first check the stability of the proposed possible configurations of SA-Cu/NG against metal aggregation at a theoretical level, in order to determine that the proposed possible catalysts are feasible experimentally (see Section S2 and Table S4 in Supplementary Materials for details). The Cu K-edge XANES curves indicate that the Cu valence of SA-Cu/NG is around +1.5 (Figure 2(c)). The oxidation state of Cu single atom is obtained for all possible models via the normalization of Bader charge to corresponding standard samples (Cu, CuO, and Cu_2_O) with known oxidation states (see Figure S19) and shown in Table S4, which can confirm whether it is consistent with XANES analysis. Besides, the bond lengths between Cu and nearest neighbor are also summarized in Table S4, which can be compared with experimental EXAFS results (Table S2, 2.02 Å). The configurations with bond length deviation beyond 10% are regarded to be inconsistent with experimental characterization. All configurations meeting the screening criteria of stability, oxidation state, and bond length are labeled in red color in S4, which are selected for further investigations on ORR theoretical activity. It should be noted that Cu-pyridine-N_4_, which is often regarded as the active center of Cu single-atom catalysts on graphene in previous works [27–29, 31, 32], is also considered here in the case, although its oxidation state (+1.94) is not in good agreement with XANES results.

To reveal the ORR electrocatalytic activity of SA-Cu/NG, the computational hydrogen electrode method is used to examine the screened active sites by DFT calculations. Generally, the ORR process through an associative mechanism involves four-electron-four-proton transfer elementary steps on isolated metal sites, generating the adsorbed OH^∗^, O^∗^, and OOH^∗^ intermediates. The optimized configurations of the intermediates and the corresponding adsorption free energies are shown in Figure S20 and Tables S5~S8. As shown in Figure S21 and Table S9, the free energy diagrams of all elementary steps in the ORR process on possible active sites of SA-Cu/NG are calculated at zero electrode potential *U* versus RHE. Among all the tested active sites of SA-Cu/NG, the Cu-2pd_N-2pd_C(side1) (marked as Cu-N_2_C_2_ in the following content) possesses the highest ORR activity, and the onset potential (*U*^onset^_RHE_) of all the tested active sites of SA-Cu/NG is apparently inferior to the onset potential 0.80 V of Pt(111). The inferior theoretical activity of all possible active sites of SA-Cu/NG to Pt(111) is entirely contradictory with the experimental results that SA-Cu/NG possesses superior ORR activity to Pt/C (Figure 3(a)). These results demonstrate that the Cu-N_2_C_2_ configuration should be the exact coordination environment of SA-Cu/NG rather than the real active species of alkaline ORR under working condition.

**Figure 4:**
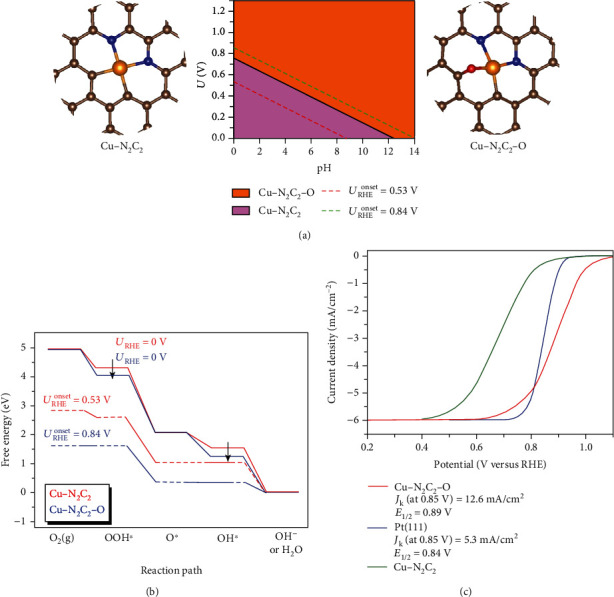
Density functional theory calculations of catalytic properties. (a) Surface Pourbaix diagrams and the configuration of Cu-N_2_C_2_ and Cu-N_2_C_2_-O. The values of onset potential are determined self-consistently. (b) Free energy for four-electron ORR on Cu-N_2_C_2_ and Cu-N_2_C_2_-O at zero electrode potential and onset electrode potential with RHE. (c) Simulated polarization curve on Pt(111), Cu-N_2_C_2_, and Cu-N_2_C_2_-O at 1600 rpm rotation speed.

Previous research has demonstrated that, under the various electrocatalytic working conditions, the adsorbate might be adsorbed at the active center and the modified surface structures would significantly boost the activity of catalysts. For example, some fresh catalytic centers of SACs (e.g., MN_4_-O and MN_4_-OH) were detected by *in situ* techniques, CHE model, and microkinetic analysis [14, 49, 50]. It can be inferred that the *in situ* generated oxygen adsorbate species may lead to reconstitutions of active sites of SA-Cu/NG and therefore enhance the ORR activity under working potential. Therefore, we systematically calculate preadsorption energies of O^∗^, OH^∗^, or OOH^∗^ intermediates on the catalytic center of SA-Cu/NG under different electrode potentials and generate the surface Pourbaix diagrams (Figure S22), from which the thermodynamically stable surface phases can be determined. As depicted in Figure S22, those active sites of SA-Cu/NG, where the Cu atoms are covered by a single O atom or a single OH at ORR working condition (at 0.7~1.23 V versus RHE) according to surface Pourbaix diagrams, are picked out for further studies on ORR theoretical activity. The adsorption free energies and reaction free energies of Cu-SACs with surface reconstitution (Figures S22c, S22e, S22g, and 22h) are shown in Tables S10~S13, and the reaction intermediates and reaction free energy along ORR are displayed in Figures S23 and S24. Among these Cu-SACs with surface reconstitution under working potential, as for Cu-N_2_C_2_-O (Figure 4(a)), unless the output potential increases to *U*_RHE_ > 0.84 V, the free energy of the first proton-electron transfer step maintains downhill, and obviously, the first step of O_2_ to form OOH^∗^ is the potential-determining step of ORR. Hence, the resultant *U*^onset^_RHE_ of Cu-N_2_C_2_-O is 0.84 V (Figure 4(b)) and higher than that for Pt(111) and Cu-N_2_C_2_. Despite the reconstituted active sites on other candidates, their ORR activities are still sluggish compared with Pt(111). There is another reaction mechanism for ORR, namely, dissociative pathway, where O_2_ is adsorbed and then dissociated into two O^∗^ species on the Cu atom. However, the dissociation energy barrier of O_2_ on SA-Cu/NG is larger than 1 eV (Figure S25), which is much higher than that of O_2_ on Pt(111) (0.48 eV) [51]. So, we believe that the associative mechanism is the most reasonable reduction pathway. To give an intuitive illustration, we further simulate their polarization curve and make a comparison with that of Pt(111) by means of microkinetic simulations (see Section 2.3 in Supplementary Materials for details). The predicted *E*_1/2_ of oxygen-reconstituted Cu-N_2_C_2_-O structure reaches ∼0.89 V versus RHE and outperforms Pt(111) by ∼50 mV (Figure 4(c)). Moreover, its *J*_k_ is larger than that of Pt(111). All theoretical outcomes show qualitatively reasonable consistency with experimental measurements, i.e., the oxygen-reconstituted structure (Cu-N_2_C_2_-O) rather than the virgin Cu-N_2_C_2_ configuration is the real active center of alkaline ORR under working potential.

### 2.5. ORR Catalytic Mechanism in the Oxygen-Reconstituted Active Species of SA-Cu/NG

**Figure 5:**
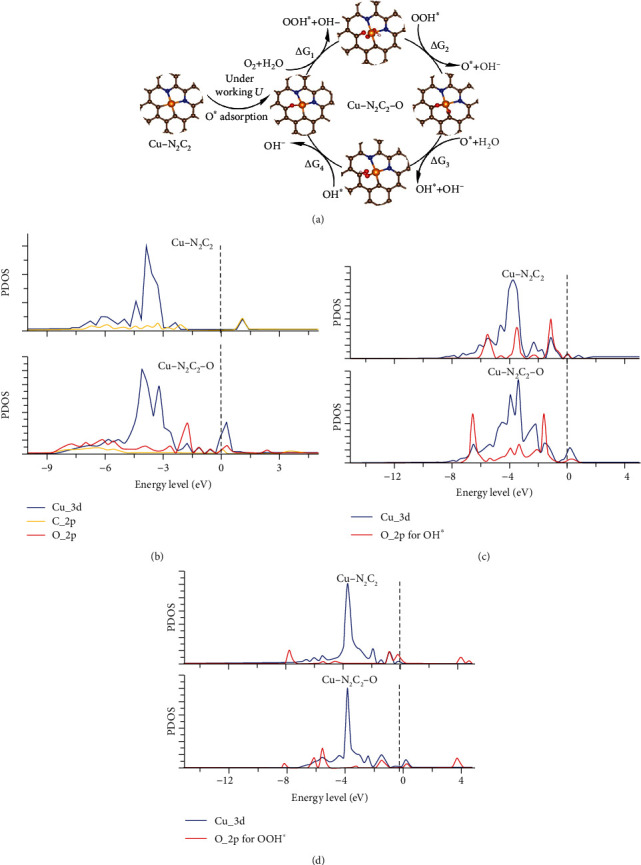
The catalytic mechanism. (a) Sketch map of the entire ORR mechanism of Cu-N-C single-atom electrocatalyst under ORR working potential. The brown, blue, red, white, and orange balls denote carbon, nitrogen, oxygen, hydrogen, and copper atoms. (b–d) Projected density of state analysis on (b) bare, (c) OH^∗^ adsorbed, (d) OOH^∗^ adsorbed Cu-2pd_N-2pd_C(side1), and Cu-2pd_N-2pd_C(side1)-O. “pd” means pyridine.

To explore the reconstituted catalytic sites and complete ORR mechanism of SA-Cu/NG under working potential, the concerning adsorption structures of the intermediate and elementary steps are shown in Figure 5(a). Under working potential, the first step involves the single oxygen adsorbed on the Cu-C bond on one side of graphene and the formation of the oxygen-reconstituted Cu-N_2_C_2_-O active center. And O_2_ reacts with water on the other side of graphene through a series of proton-coupled electron transfer, finally resulting in the release of OH^−^. Actually, the adsorption or reaction process on both sides of SA-Cu/NG is rational, as confirmed in other SACs by previously reported *in situ* EXAFS [49, 52]. The enhanced ORR activity from Cu-N_2_C_2_ to Cu-N_2_C_2_-O derives from the increased affinity of Cu atom for intermediates. Compared with Cu-N_2_C_2_, Cu atom of Cu-N_2_C_2_-O breaks up one Cu-C bond to form a three-coordination configuration when binding either OOH^∗^ or OH^∗^. And the adsorption strength becomes stronger making the process of forming OOH^∗^ and OH^∗^ much more exothermic, which yields a higher onset potential.  Figure 5(b) implies the important role of extraneous oxygen atom adsorption in weakening Cu-C bond strength through bringing down the energy level of antibonding orbital. To understand the effect of reduced coordination number of Cu atom on higher stability of OOH^∗^ and OH^∗^ on Cu-N_2_C_2_-O relative to Cu-N_2_C_2_, it is valuable to probe the d-orbital Cu site contributing to ORR. With the upward shifting of d-band of Cu atom from Cu-N_2_C_2_ to Cu-N_2_C_2_-O (see Figures 5(c) and 5(d)), the antibonding orbit upshifts, bringing about the reduced antibond orbit occupation and more robust adsorption for OOH^∗^ and OH^∗^. The same phenomenon, that the introduction of oxygen atom reduces the coordination number of Cu atom when binding OOH^∗^ and OH^∗^ and yields the higher adsorption strength, is also observed in other candidate active sites of SA-Cu/NG (Figure S23 and Tables S11 and S12). Thus, the above DFT provides strong analysis to verify the critical effect of the CuN_2_C_2_ configuration as well as oxygen reconstruction during operando operation for enhancing the ORR performance of SA-Cu/NG.

### 2.6. Acidic ORR Tests

ORR performance at acidic condition for SA-Cu/NG is further evaluated. As seen in Figure S26, *E*_1/2_ of SA-Cu/NG at acidic condition is only 0.48 V (versus RHE) that is about 320 mV inferior to Pt/C in 0.5 M H_2_SO_4_ solution. The rather sluggishness performance of SA-Cu/NG for ORR under acidic condition is in line with the inferior theoretical half-wave potential of Cu-N_2_C_2_ to Pt(111) in Figure 4(c), which implies that the virgin activity sites (Cu-N_2_C_2_) rather than the oxygen-reconstituted active species (Cu-N_2_C_2_-O) of SA-Cu/NG are responsible for the inferior activity of SA-Cu/NG at acidic medium. In short, the finding of oxygen-reconstituted active species of SA-Cu/NG at alkaline media can successfully unveil the bottleneck puzzle of why the performance of ORR catalysts under alkaline condition is better than that under acidic condition.

## 3. Conclusions

In summary, we have synthesized a novel single-atom Cu catalyst (SA-Cu/NG) with Cu-N_2_C_2_ configuration, and SA-Cu/NG shows the superior ORR ability to Pt/C under alkaline medium while inferior activity to 20% Pt/C at acidic condition. Together with experimental data and DFT theoretical analysis, we for the first time found that the oxygen-reconstituted Cu-N_2_C_2_-O structure under working condition is the realistic ORR active species at alkaline conditions, while the virgin Cu-N_2_C_2_ structure is the ORR active moiety at acidic media. The theoretical half-wave potentials of both oxygen-reconstituted Cu-N_2_C_2_-O structure and virgin Cu-N_2_C_2_ structure are also in perfect agreement with experimental results at alkaline and acidic media, respectively. Adsorption of atomic oxygen intermediate O^∗^ on the Cu-C site yielded the reconstituted structure, and such O^∗^ adsorption optimizes the electronic structure of Cu atom by breaking one Cu-C bond during binding of OH^∗^ and OOH^∗^ reaction intermediates and therefore boosts ORR activity. The finding of reconstitution of surface active sites unveils the bottleneck puzzle of why the performance of ORR catalysts under alkaline electrolyte is better than that under acidic condition, which also offers a fresh perspective to advisably fabricate high-performance ORR electrocatalysts.

## 4. Materials and Methods

Experimental details including synthesis of samples, structure, and electrochemical characterizations of SA-Cu/NG and other samples and performance of SA-Cu/NG-based Zn-air batteries are presented in Supplementary Materials. All computational details, including the DFT calculation process, screening of all possible structures of Cu single-atom catalysts, free energy diagram of Cu-SAC, and surface-reconstituted Cu-SACs and surface Pourbaix diagrams of Cu-SACs, are also described in Supplementary Materials.

## Supplementary Materials

Supplementary Materials Section S1: experimental section. Section S2: density functional theory calculations. Section S3: supporting figures. Figure S1: XRD of SA-Cu/NG and NG. Figure S2: HAADF STEM of the SA-Cu/NG. (b) HRTEM of SA-Cu/NG. Figure S3: XPS of SA-Cu/NG and NG. Figure S4: N 1s XPS of SA-Cu/NG. Figure S5: N 1s XPS of NG. Figure S6: Cu 2p XPS of SA-Cu/NG. Figure S7: N_2_ adsorption and desorption isotherms for SA-Cu/NG and NG. Figure S8: the pore size of SA-Cu/NG and NG. Figure S9: *J*_k_ of SA-Cu/NG, NG, and 20% Pt/C in alkaline media. Figure S10: LSV of (a) SA-Cu/NG and (c) NG under 0.1 M KOH condition. K-L curves of (b) SA-Cu/NG and (d) NG. Figure S11: (a) LSV and (b) K-L curves of 20% Pt/C under 0.1 M KOH solution. Figure S12: *n* from 0.2 V to 0.8 V for SA-Cu/NG, NG, and 20% Pt/C in alkaline media. Figure S13: *i*–*t* measurement of SA-Cu/NG and 20% Pt/C in alkaline media at 0.6 V (versus RHE). Figure S14: *i*–*t* measurement of SA-Cu/NG and 20% Pt/C by dropping CH_3_OH after ~400 s. Figure S15: open circuit voltage of SA-Cu/NG assembled battery is measured by a universal meter. Figure S16: the stability test for SA-Cu/NG+IrO_2_-based battery for 90 h. Figure S17: the picture of LED lighted by three SA-Cu/NG+IrO_2_-based batteries. Figure S18: top view of all possible structures of Cu SACs. “pd” means pyridine, and “pr” means pyrrole. Figure S19: the scaling relationship between electron depletion of Cu atoms and valance state of Cu. Figure S20: top view of relaxed configurations of intermediate along ORR adsorbed on Cu SACs. “pd” means pyridine, and “pr” means pyrrole. Figure S21: free energy diagram for 4e- transfer ORR on Cu single-atom catalysts at zero electrode potential and onset electrode potential with reversible hydrogen electrode (RHE) in an alkaline electrolyte. “pd” means pyridine, and “pr” means pyrrole. Figure S22: surface Pourbaix diagrams of Cu SACs. The values of onset potential are determined self-consistently. Figure S23: top view of relaxed configurations of intermediate along ORR adsorbed on intermediate-modified Cu single-atom catalysts. Figure S24: free energy diagram for 4e- transfer ORR on Cu single-atom catalysts at zero electrode potential and onset electrode potential with reversible hydrogen electrode (RHE) in an alkaline electrolyte. “pd” means pyridine, and “pr” means pyrrole. Figure S25: free energy diagram for O_2_ dissociation pathway on Cu-4pd_N, Cu-2pd_N-2pd_C-side1, and Cu-2pd_N-2pd_C-side1-O to form two adsorbed O^∗^. “pd” means pyridine, and “pr” means pyrrole. Figure S26: (a) LSV curves of SA-Cu/NG and 20% Pt/C in 0.5 M H_2_SO_4_ media. Section S4: supporting tables. Table S1: summary of XPS data of SA-Cu/NG and NG. Table S2: parameters of EXAFS fit for SA-Cu/NG. Table S3: the porosity of SA-Cu/NG and NG. Table S4: the valance of Cu atom, antiaggregation energy, antileach energy, and bond length of Cu-neighbor atom for each possible active center of different Cu SACs shown in Figure S19. Table S5: values used for the corrections in determining the free energy of free H_2_ and H_2_O molecule. Table S6: DFT adsorption energies (Δ*E*_ad_, eV), zero-point energy (ZPE, eV), zero-point energy correction (*Δ*ZPE, eV), entropy (*T*∗*S*, eV, *T* = 298 K), entropy correction (*T*∗Δ*S*, eV, *T* = 298 K), and adsorption free energies (Δ*G*_ad_, eV) of the adsorbed O along the reaction pathway of ORR on different Cu SACs. Adsorption energies (eV) were taken relative to energies of free H_2_ and H_2_O molecules. Table S7: DFT adsorption energies (Δ*E*_ad_, eV), zero-point energy (ZPE, eV), zero-point energy correction (*Δ*ZPE, eV), entropy (*T*∗*S*, eV, *T* = 298 K), entropy correction (*T*∗Δ*S*, eV, T = 298 K), and adsorption free energies (ΔG_ad_, eV) of the adsorbed OH along the reaction pathway of ORR on different Cu SACs. Adsorption energies (eV) were taken relative to energies of free H_2_ and H_2_O molecules. Table S8: DFT adsorption energies (Δ*E*_ad_, eV), zero-point energy (ZPE, eV), zero-point energy correction (*Δ*ZPE, eV), entropy (*T*∗*S*, eV, *T* = 298 K), entropy correction (*T*∗Δ*S*, eV, *T* = 298 K), and adsorption free energies (Δ*G*_ad_, eV) of the adsorbed OOH along the reaction pathway of ORR on different Cu SACs. Adsorption energies (eV) were taken relative to energies of free H_2_ and H_2_O molecules. Table S9: reaction free energy (in eV vs. RHE) of elementary step at *U*_RHE_ = 0 V and onset potential (in V vs. RHE) for ORR on different Cu SACs. Table S10: DFT adsorption energies (Δ*E*_ad_, eV), zero-point energy (ZPE, eV), zero-point energy correction (*Δ*ZPE, eV), entropy (*T*∗*S*, eV, *T* = 298 K), entropy correction (*T*∗Δ*S*, eV, *T* = 298 K), and adsorption free energies (Δ*G*_ad_, eV) of the adsorbed O along the reaction pathway of ORR on O-reconstituted Cu SACs. Adsorption energies (eV) were taken relative to energies of free H_2_ and H_2_O molecules. Table S11: DFT adsorption energies (Δ*E*_ad_, eV), zero-point energy (ZPE, eV), zero-point energy correction (*Δ*ZPE, eV), entropy (*T*∗*S*, eV, *T* = 298 K), entropy correction (*T*∗Δ*S*, eV, *T* = 298 K), and adsorption free energies (Δ*G*_ad_, eV) of the adsorbed OH along the reaction pathway of ORR on O-reconstituted Cu SACs. Adsorption energies (eV) were taken relative to energies of free H_2_ and H_2_O molecules. Table S12: DFT adsorption energies (Δ*E*_ad_, eV), zero-point energy (ZPE, eV), zero-point energy correction (*Δ*ZPE, eV), entropy (*T*∗*S*, eV, *T* = 298 K), entropy correction (*T*∗Δ*S*, eV, *T* = 298 K), and adsorption free energies (Δ*G*_ad_, eV) of the adsorbed OOH along the reaction pathway of ORR on O-reconstituted Cu SACs. Adsorption energies (eV) were taken relative to energies of free H_2_ and H_2_O molecules. Table S13: reaction free energy (in eV vs. RHE) of elementary step at *U*_RHE_ = 0 V and onset potential (in V vs. RHE) for ORR on different O-reconstituted Cu SACs.
