# Supplementary material for: Oxygen-Reconstituted Active Species of Single-Atom Cu Catalysts for Oxygen Reduction Reaction
Source: Research (Wash D C). 2020 Oct 5;2020:7593023. doi: 10.34133/2020/7593023 (PMC7556117; doi:10.34133/2020/7593023)
Supplement: Supplementary Materials — Section S1: experimental section. Section S2: density functional theory calculations. Section S3: supporting figures. Figure S1: XRD of SA-Cu/NG and NG. Figure S2: HAADF STEM of the SA-Cu/NG. (b) HRTEM of SA-Cu/NG. Figure S3: XPS of SA-Cu/NG and NG. Figure S4: N 1s XPS of SA-Cu/NG. Figure S5: N 1s XPS of NG. Figure S6: Cu 2p XPS of SA-Cu/NG. Figure S7: N2 adsorption and desorption isotherms for SA-Cu/NG and NG. Figure S8: the pore size of SA-Cu/NG and NG. Figure S9: Jk of SA-Cu/NG, NG, and 20% Pt/C in alkaline media. Figure S10: LSV of (a) SA-Cu/NG and (c) NG under 0.1 M KOH condition. K-L curves of (b) SA-Cu/NG and (d) NG. Figure S11: (a) LSV and (b) K-L curves of 20% Pt/C under 0.1 M KOH solution. Figure S12: n from 0.2 V to 0.8 V for SA-Cu/NG, NG, and 20% Pt/C in alkaline media. Figure S13: i–t measurement of SA-Cu/NG and 20% Pt/C in alkaline media at 0.6 V (versus RHE). Figure S14: i–t measurement of SA-Cu/NG and 20% Pt/C by dropping CH3OH after ~400 s. Figure S15: open circuit voltage of SA-Cu/NG assembled battery is measured by a universal meter. Figure S16: the stability test for SA-Cu/NG+IrO2-based battery for 90 h. Figure S17: the picture of LED lighted by three SA-Cu/NG+IrO2-based batteries. Figure S18: top view of all possible structures of Cu SACs. “pd” means pyridine, and “pr” means pyrrole. Figure S19: the scaling relationship between electron depletion of Cu atoms and valance state of Cu. Figure S20: top view of relaxed configurations of intermediate along ORR adsorbed on Cu SACs. “pd” means pyridine, and “pr” means pyrrole. Figure S21: free energy diagram for 4e- transfer ORR on Cu single-atom catalysts at zero electrode potential and onset electrode potential with reversible hydrogen electrode (RHE) in an alkaline electrolyte. “pd” means pyridine, and “pr” means pyrrole. Figure S22: surface Pourbaix diagrams of Cu SACs. The values of onset potential are determined self-consistently. Figure S23: top view of relaxed configurations of intermediate alon [file 7593023.f1.docx]

**Supporting Information**

**Oxygen-reconstituted active species of single-atom Cu catalysts for oxygen reduction reaction**

Liu Yang^a,1^, Haoxiang Xu^a,1^, Huibing Liu^a,1^, Xiaofei Zeng^a^, Daojian Cheng^a^, Yan Huang^a^, Lirong Zheng^b^, Rui Cao^c^ and Dapeng Cao^a,*^

^a^ Beijing Advanced Innovation Center for Soft Matter Science and Engineering, State Key Laboratory of Organic-Inorganic Composites, Beijing University of Chemical Technology, Beijing 100029, People’s Republic of China

^b^ Beijing Synchrotron Radiation Facility, Institute of High Energy Physics, Chinese Academy of Sciences, Beijing 100049, China

^c^ Stanford Synchrotron Radiation Lightsource, SLAC National Accelerator Laboratory, Menlo Park, California 94025, United States

^1^ Equally contributed to this work

*Corresponding author. Email: [caodp@mail.buct.edu.cn](mailto:caodp@mail.buct.edu.cn)

**Section S1: Experimental section**

**S1.1 Reagent**

Urea was bought from Alfar Aesar, copper nitrate hydrate, polyether F127 and dopamine hydrochloride were purchased from MACKLIN and HCl were bought from Beijing Chemical Works. All chemicals in the synthesis are analytical reagents without further treatment.

**S1.2 Synthesis of SA-Cu/NC**

0.2 g g-C_3_N_4_ and 0.2 g F127 were distributed in 30 ml deionized water and sonicated. Next, 0.027 mmol copper nitrate hydrate and 0.075 g dopamine hydrochloride were mixed in the abovementioned mixture and agitated 24 h. Then evaporate the solution above. The dry was pyrolyzed at 550℃ 2h and then heated to 800℃ 2 h in N_2_ atmosphere. The pyrolyzed sample was leached with HCl, then the sample experienced secondary carbonization, which denoted as SA-Cu/NC. For comparison, under the same conditions of other synthesis steps, except for lacking addition of Cu metal, the final sample was obtained that was marked as NG sample.

**S1.3 Materials characterization**

SEM and TEM images were gained from a S4700 SEM and Hitachi-7700 TEM instrument, respectively. The high-resolution TEM, HAADF-STEM image were obtained from FEI Themis G2 with double Cs corrector. The specific surface area was tested through N_2_ adsorption-desorption isotherms applying an ASAP 2460 analyzer (Micromeritics, U.S.A). XRD were tested on a D/MAX 2000 X-ray diffractometer. XPS was conducted on ThermoVG ESCALAB 250 with an A1 Kα as X-ray source. The XAFS curves of Cu K-edge were performed at 1W1B station (Beijing Synchrotron Radiation Facility). The analysis of EXAFS data were conducted using IFEFFIT software packages.

**S1.4 Electrochemical test for ORR**

The test were evaluated on the CHI760e workstation using three electrode setup. The prepared catalyst was served as working electrode, Pt/C was used as a counter electrode, and a saturated calomel was regarded as a reference electrode. All potentials are corrected by the Nernst equation. Further, 5 mg synthesized samples and 50 μl 5 wt% Nafion was spread evenly in 700 μl deionized water and 300 μl ethanol through ultrasound. Then, the catalyst inks (10 ml) were dropped onto a glassy carbon electrod to assess the electrochemical activity of the electrocatalysts. Linear sweep voltammetry (LSV) was obtained at 1600 rpm in 0.1 M KOH or 0.5 M H_2_SO_4_ electrolyte. The transfer electron number n and the H_2_O_2_ yield were obtained in view of the below equations:

$$n=4\times\frac{I_{\mathrm{disk}}}{\left( \frac{I_{\mathrm{ring}}}{N} \right)+I_{\mathrm{disk}}}$$

$$H_{2}O_{2}\left( \% \right)=200\times\frac{I_{\mathrm{ring}}/N}{\left( \frac{I_{\mathrm{ring}}}{N} \right)+I_{\mathrm{disk}}}$$

where I_disk_ is the disk current, I_ring_ is the ring current, N is the current efficiency of Pt ring. N value is set to 0.42.

n can also be calculated by Koutecky–Levich equation:

$$\frac{1}{\text{J}}=\frac{1}{\text{J}_{\text{L}}}+\frac{1}{\text{J}_{\text{K}}}=\frac{1}{\text{J}_{\text{K}}}+\frac{1}{\text{B}\omega^{1/2}}$$

$$\text{B}=0.62nFC_{0}D^{2/3}\upsilon^{-1/6}$$

where J, J_k_, J_L_ are the measured, kinetic and limiting current densities. $\omega$ is the angular velocity. F is the Faraday constant. C_0_ is the bulk concentration of O_2_ (1.2×10^-6^ mol/cm^3^). D is the diffusion modulus of O_2_ (cm^2^/s). $\upsilon$ is the electrolyte viscosity.

**S1.5 Zn-air assembly**

The Zn-air battery was used a Zn foil and SA-Cu/NG (1 mg/cm^2^) served as the anode and cathode. The electrolyte was 6 M KOH and 0.2 M ZnO. Further, the stability of SA-Cu/NG+IrO_2_ was served as air cathode for recyclable Zn-air battery.

**Section S2. Density Functional Theory Calculations**

**S2.1 The circumscription of the thermodynamic stability of the SA-Cu/NG**

*The definition of the anti-leach energy of the* *SA-Cu/NG*

We check for the stability of the *SA-Cu/NG* preventing the leaching of metal is the anti-leaching energy of the metal atoms on the carbon matrix.

△E_anti-leach energy_ = E_graphene_ + E(Cu^2+^(aq)) – E_graphene+atom_

E(Cu^2+^(aq)) = E_bulk_ – 2eU_0_, U_0_ = + 0.3419 V

If △E_anti-leach energy_ < 0, we assume that Cu SAC embedded into the graphene occurs prior to metal being leached.

*The definition of the anti-aggregation energy of the Cu SAC*

Another descriptor is the detection of Cu SAC against metal atom aggregation that is the anti-aggregation energy, as defined below.

E_coh_ = E_bulk_- *4**E_free-atom_ (*4* is the number of Cu atom in bulk)

E_ad_ = E_graphene+atom_- E_graphene_- E_free-atom_

△E_anti-aggregation energy_ = E_ad_ − E_coh_

If △E_anti-aggregation energy_ < 0, we assume that Cu SAC embedded into the graphene occurs prior to metal aggregation.

**S2.2 Surface Pourbaix diagrams**

To determine the relative stability on different surfaces termination, we adopted the chemical potentials μ of the H_2_O and H_2_ adsorbate.

μ_O_ = [G_H2O_ − G_H2_] + 2⨯[eU − ΔG_H+_(pH)]

μ_OH_ = [G_H2O_ – 1/2⨯G_H2_] + [eU − ΔG_H+_(pH)]

μ_OOH_ =[2⨯G_H2O_ – 3/2⨯G_H2_] + [eU − ΔG_H+_(pH)]

We adopted the definition of reaction free energy of adsorption process of reaction species including O^*^, OH^*^ and OOH^*^, which can be obtained as ΔG = G_slab+adsorbates_ − G_slab_ − μ_adsorbate_.

**S2.3 Polarization Curve Simulation**

Based on the kinetic analysis model of oxygen reduction reaction proposed by Nørskov et al., we simulated the polarization curve of Cu SACs. Oxygen arrive to the catalyst surface.

$O_{2}\left( aq \right)\underset{\leftrightarrow}{k1}O_{2}\left( dl \right)$ (S1)

where O_2_(aq) and O_2_(dl) represent O_2_ in the bulk electrolyte and catalyst-electrolyte interface. Next, O_2_ may adsorb on Cu SACs.

${*+ O}_{2}\left( dl \right)\underset{\leftrightarrow}{k2}O_{2}^{*}$ (S2)

Once adsorbed on Cu SACs, O_2_ forms H_2_O via electron transfer procedures, the electrochemical reaction steps are listed by the following equations:

$O_{2}^{*}+H^{+}+ e^{-}\underset{\leftrightarrow}{k3} {OOH}^{*} (acidic \mathrm{electrolyte})$

$O_{2}^{*}+H_{2}O+ e^{-}\underset{\leftrightarrow}{k3} {OOH}^{*}+{OH}^{-} (alkaline \mathrm{electrolyte})$ (S3)

${OOH}^{*}+H^{+}+ e^{-}\underset{\leftrightarrow}{k4} O^{*}+ H_{2}O$ $(acidic \mathrm{electrolyte})$

${OOH}^{*}+e^{-}\underset{\leftrightarrow}{k4} O^{*}+{OH}^{-}$ $(alkaline \mathrm{electrolyte})$ (S4)

$O^{*}+H^{+}+ e^{-}\underset{\leftrightarrow}{k5} {OH}^{*}$ $(acidic \mathrm{electrolyte})$

$O^{*}+H_{2}O+ e^{-}\underset{\leftrightarrow}{k5} {OH}^{*}+{OH}^{-} (alkaline \mathrm{electrolyte})$ (S5)

${OH}^{*}+H^{+}+ e^{-}\underset{\leftrightarrow}{k6} *+ H_{2}O$ $(acidic \mathrm{electrolyte})$

${OH}^{*}+ e^{-}\underset{\leftrightarrow}{k6} {*+OH}^{-} (alkaline \mathrm{electrolyte})$ (S6)

According to the above reduction steps, we can gained the rate equations of each absorbates as follow.

$\frac{\partial x_{O_{2}(dl)}}{\partial t}= k_{1}^{+}x_{O_{2}(aq)}-k_{1}^{-}x_{O_{2}\left( dl \right)}-k_{2}^{+}x_{O_{2}\left( dl \right)}\theta_{*}+k_{2}^{-}\theta_{O_{2}^{*}}$

$$\frac{\partial\theta_{O_{2}^{*}}}{\partial t}= k_{2}^{+}x_{O_{2}\left( dl \right)}\theta_{*}-k_{2}^{-}\theta_{O_{2}^{*}}- k_{3}^{+}\theta_{O_{2}^{*}}+ k_{3}^{-}\theta_{{OOH}^{*}}$$

$$\frac{\partial\theta_{{OOH}^{*}}}{\partial t}= k_{3}^{+}\theta_{O_{2}^{*}}- k_{3}^{-}\theta_{{OOH}^{*}}- k_{4}^{+}\theta_{{OOH}^{*}}+ k_{4}^{-}\theta_{O^{*}}$$

$$\frac{\partial\theta_{O^{*}}}{\partial t}= k_{4}^{+}\theta_{{OOH}^{*}}-k_{4}^{-}\theta_{O^{*}}- k_{5}^{+}\theta_{O^{*}}+ k_{5}^{-}\theta_{{OH}^{*}}$$

$$\frac{\partial\theta_{{OH}^{*}}}{\partial t}= k_{5}^{+}\theta_{O^{*}}- k_{5}^{-}\theta_{{OH}^{*}}- k_{6}^{+}\theta_{{OH}^{*}}+ k_{6}^{-}\theta_{*}x_{H_{2}O}$$

In these equations $x_{H_{2}O}=1$ and $x_{O_{2}(aq)}=2.34*{10}^{-5}$ corresponding to 1 atm O_2_(g) in equilibrium with O_2_(aq). Additionally, site conservation on the Cu atom of Cu SACs must be satisfied the following equation

$$\theta_{O^{*}}+\theta_{O^{*}}+\theta_{{OH}^{*}}+\theta_{{OOH}^{*}}+\theta_{O_{2}^{*}}=1$$

As for non-electrochemical reaction steps, its rate constant (k_i_) is

$$k_{i}=v_{i}exp(-\frac{E_{a,i}}{k_{B}T})$$

where v_i_ is the pre-exponential factor taken as 8⨯10^5^ an 10^9^ for S1 and S2, respectively. And E_a,i_ is the activation energy taken as 0 eV for both S1 and S2. K_B_ is the Boltzmann constant.

While as to electrochemical reaction steps, the rate constants is written as

$$k_{i}=A_{i}exp(-\frac{E_{a,i}}{k_{B}T})exp(-\frac{e\beta_{i}(U-U_{i})}{k_{B}T})$$

where A_i_ is the factor, β_i_ is the symmetric factor, E_a,i_ is the activation energy. Following previous research, we suppose A_i_, βi and E_a,i_ are unrelated with material. A_i_=10^9^, βi = 0.5 and E_a,i_ = 0.26 eV.

The k for reverse reaction, k_-i_, are calculated from

$$k_{-i}=\frac{k_{i}}{K_{i}}$$

Ki is the equilibrium constant.

$$K_{i}=exp(-\frac{\Delta G_{i}}{k_{B}T})$$

where ΔG_i_ for electrochemical reaction steps is depends on the following equation

$$\Delta G_{i}=\Delta G_{i}^{0}+eU$$

ΔG_i_^0^ is 0 V(*versus*, RHE) free energy, which are listed in Table S9 and S13, and U is the potential *versus*, RHE.

Finally, current density (j) can be calculated by

j= eρTOF_e-_

where e= 1.6×10^-19^C and ρ=80.3 μC/cm^2^.

**S2.4 DFT computational details**

All DFT calculations with consideration of spin-polarization were conducted in the VASP code[^1^](#_ENREF_1)^,^ [^2^](#_ENREF_2). Meanwhile, we used the Perdew-Burke-Ernzerhof functional to depict the exchange energy[^3^](#_ENREF_3). We used the PAW pseudo-potentials to depict the interaction between valence electron and ionic cores[^4^](#_ENREF_4). We used VASPsol to solve solvent effect.[^5^](#_ENREF_5) The ORR free energy calculation on atomically dispersed Cu catalysts supported on graphene were calculated according to electrochemical framework of calculation hydrogen electrode developed by Nørskov, which can be referred to our previous works.[^6^](#_ENREF_6)^,^ [^7^](#_ENREF_7)

**Section S3: Supporting Figures**

**
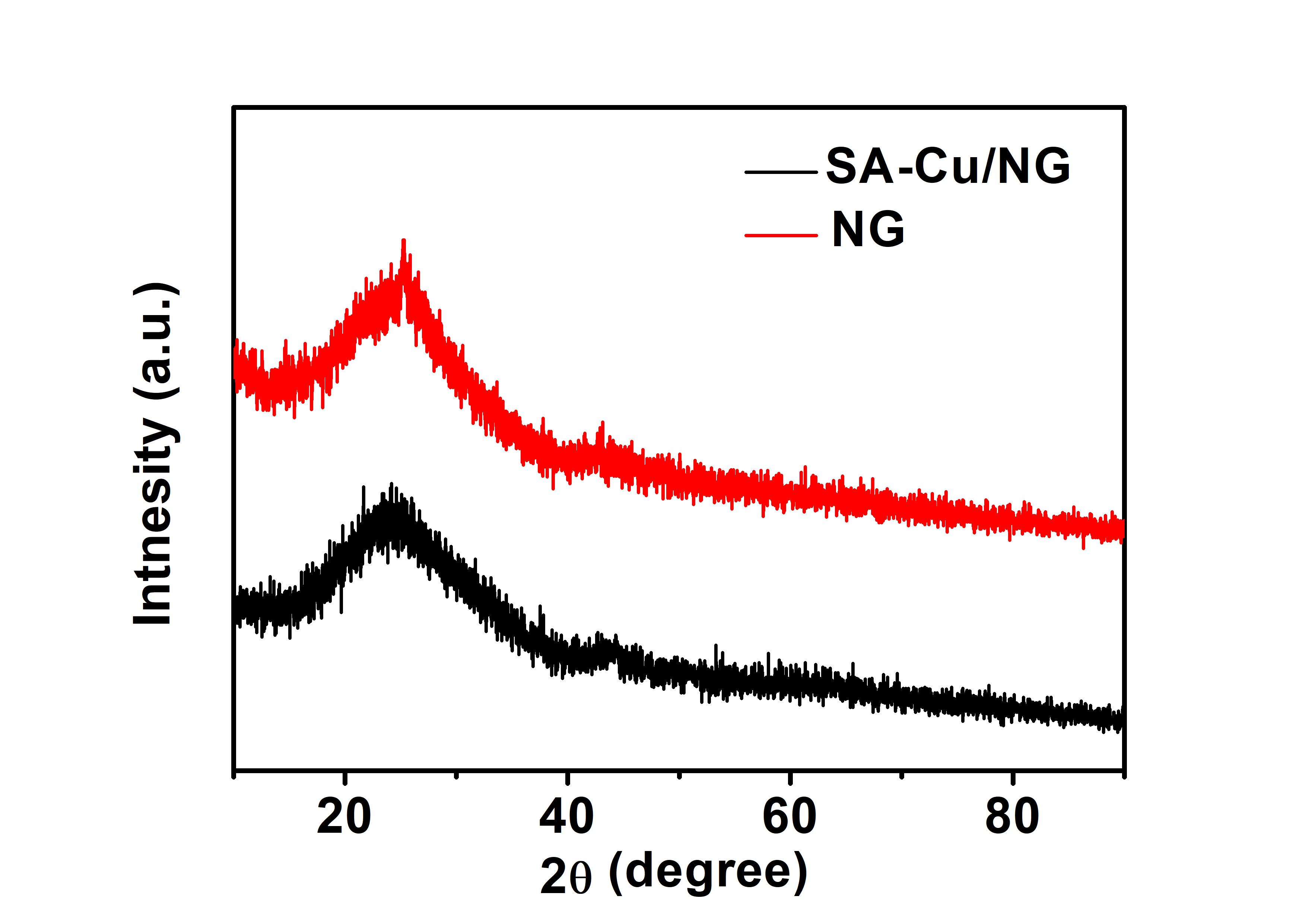
**

**Figure S1** XRD of SA-Cu/NG and NG.

**
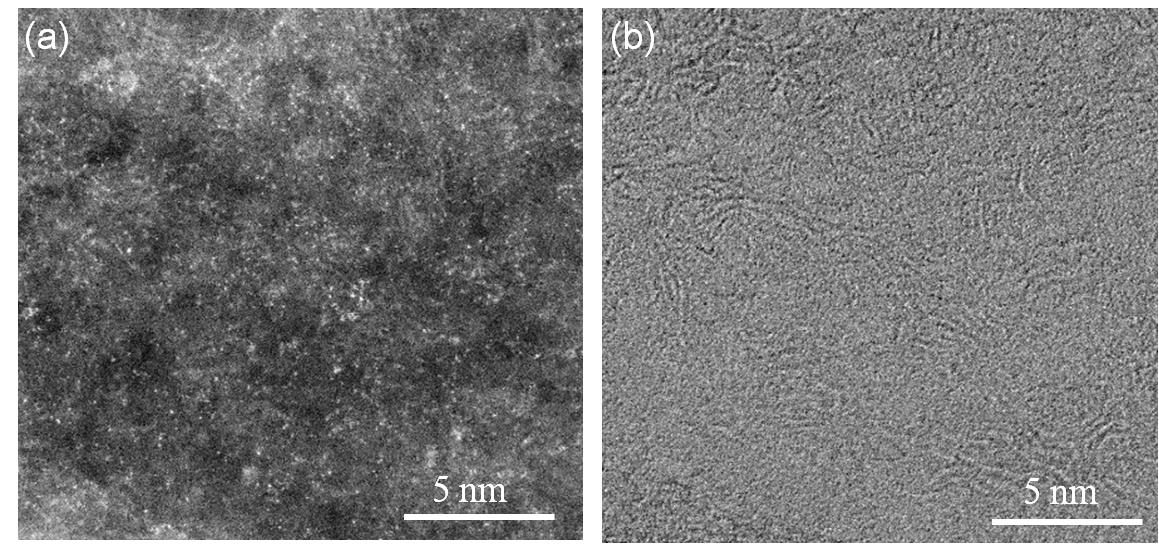
**

**Figure S2** HAADF STEM of the SA-Cu/NG. (b) HRTEM of SA-Cu/NG.

**
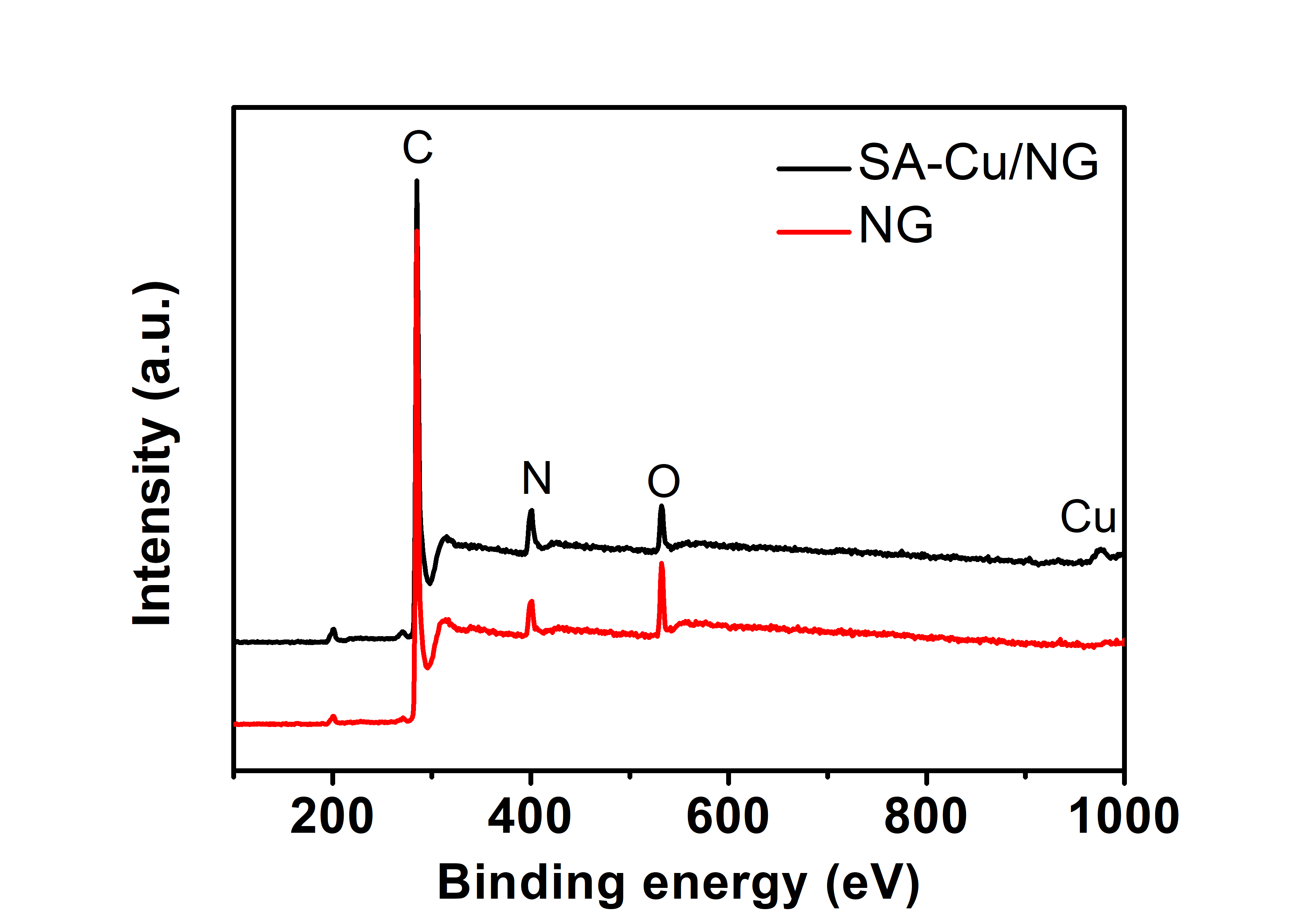
**

**Figure S3** XPS of SA-Cu/NG and NG.


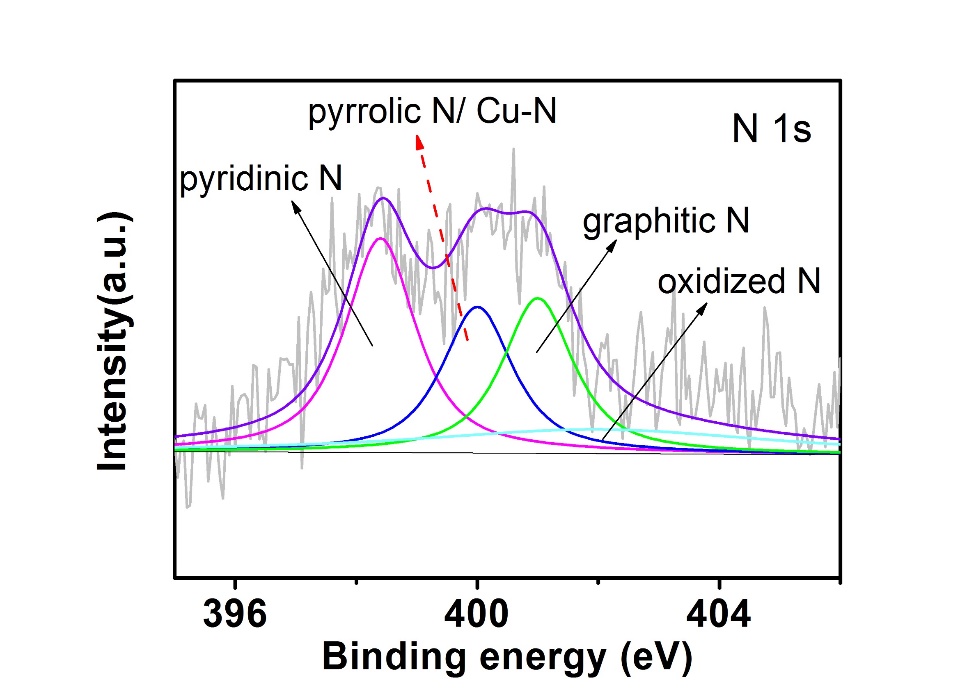


**Figure S4** N 1s XPS of SA-Cu/NG.

**
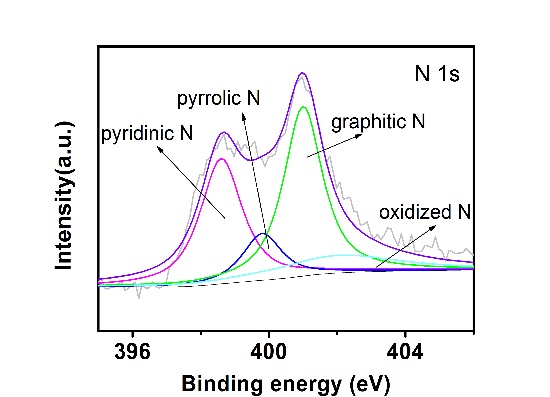
**

**Figure S5** N 1s XPS of NG.


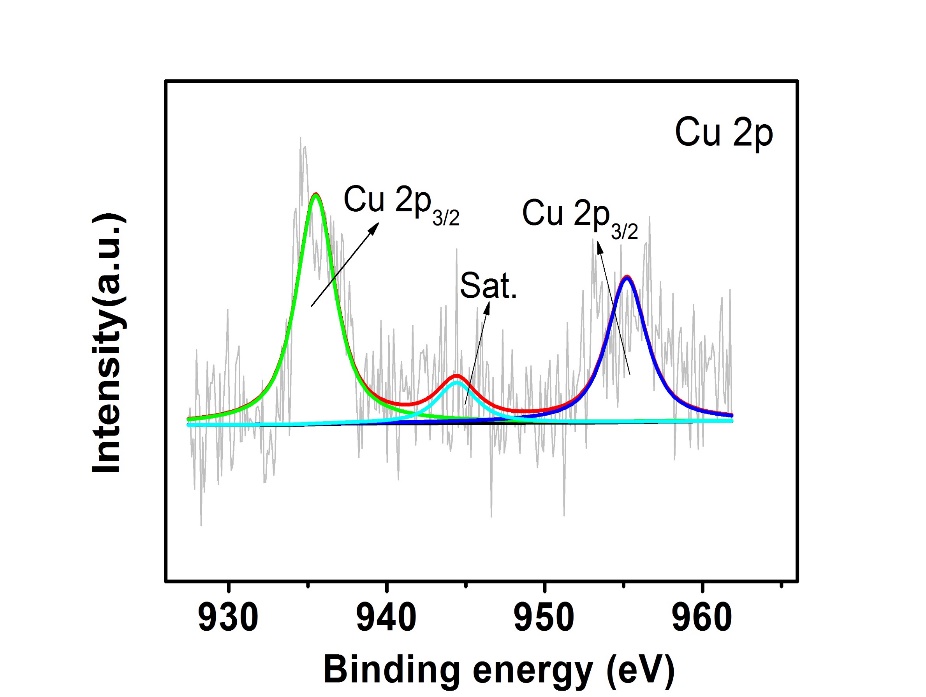


**Figure S6** Cu 2p XPS of SA-Cu/NG.
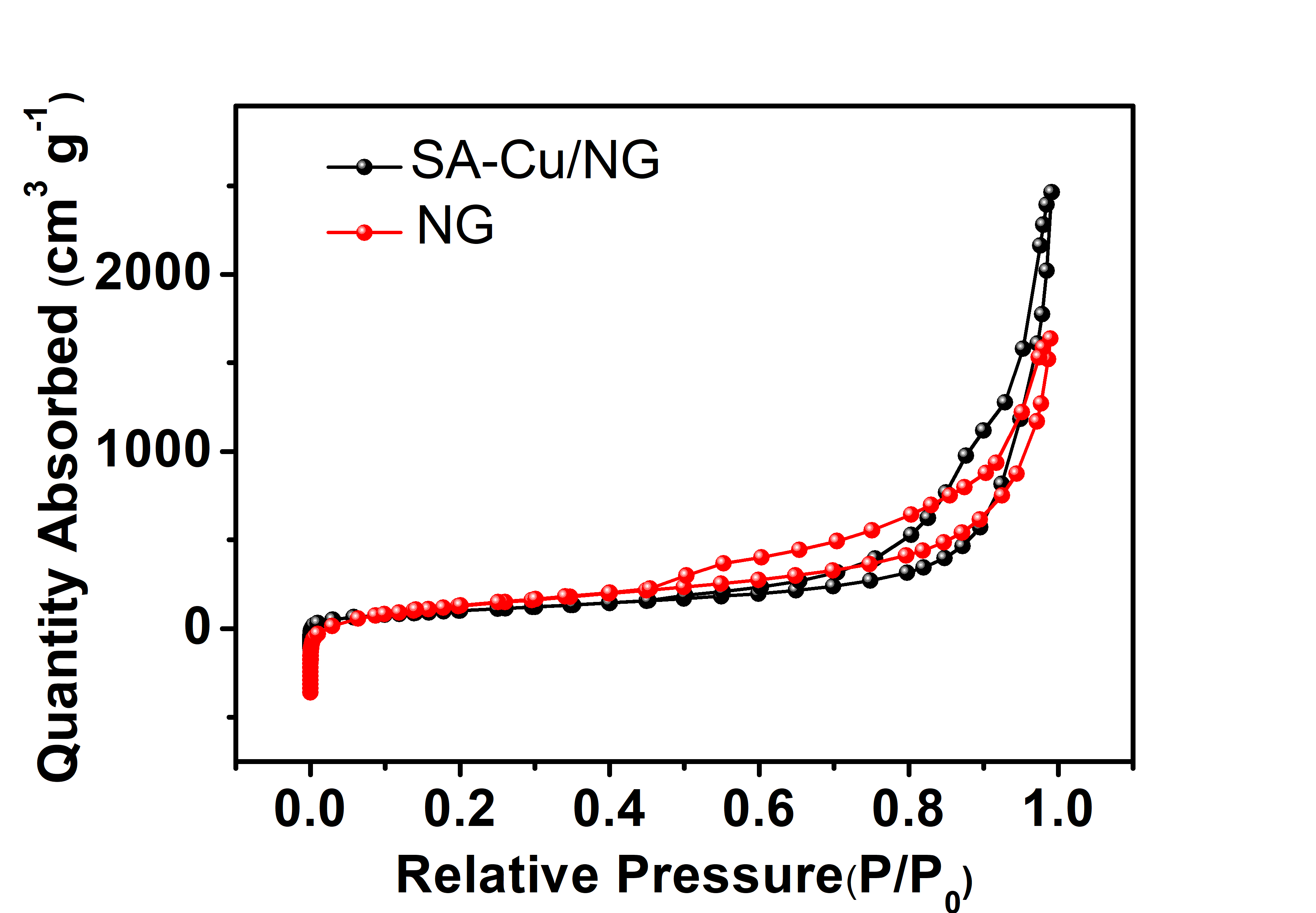


**Figure S7** N_2_ adsorption and desorption isotherms for SA-Cu/NG and NG.

**
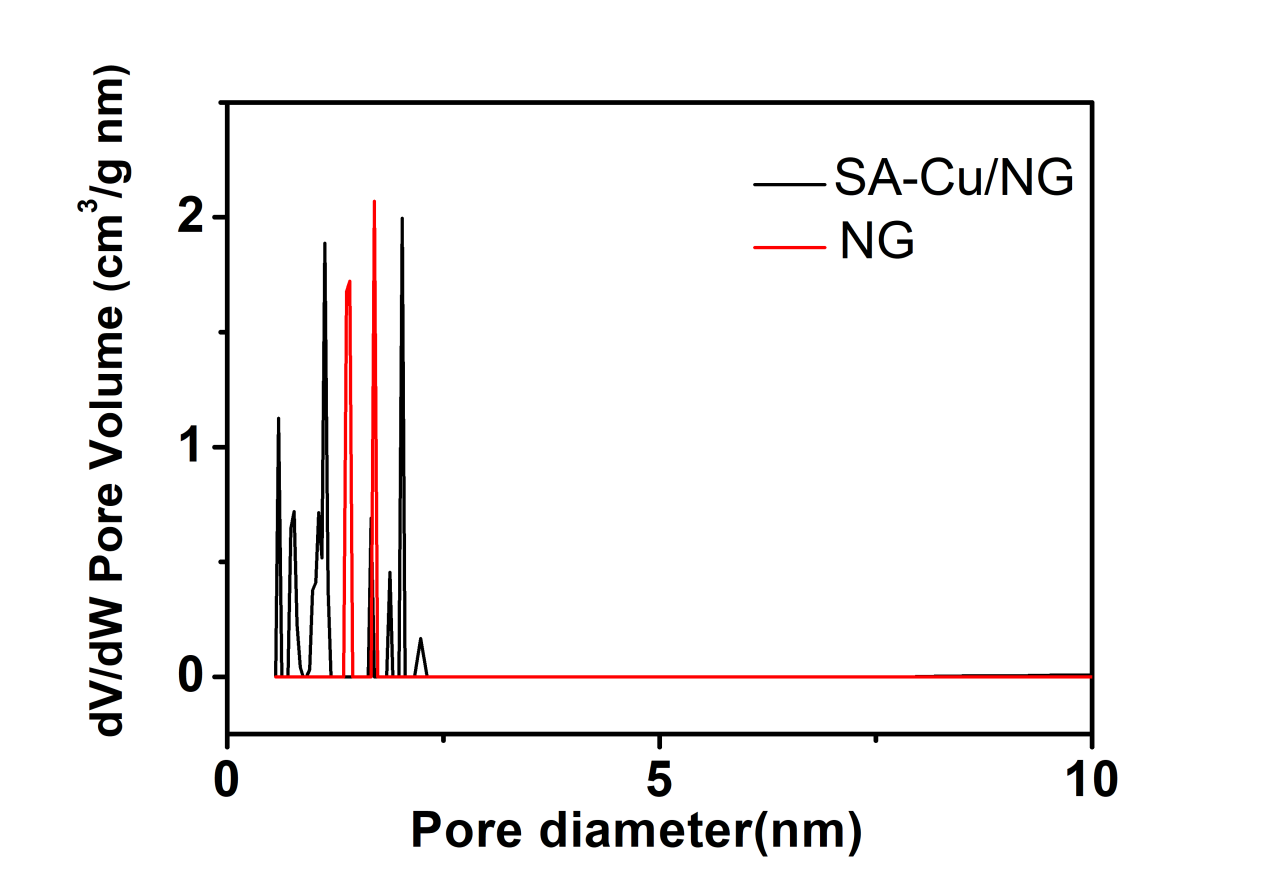
**

**Figure S8** The pore size of SA-Cu/NG and NG.

**
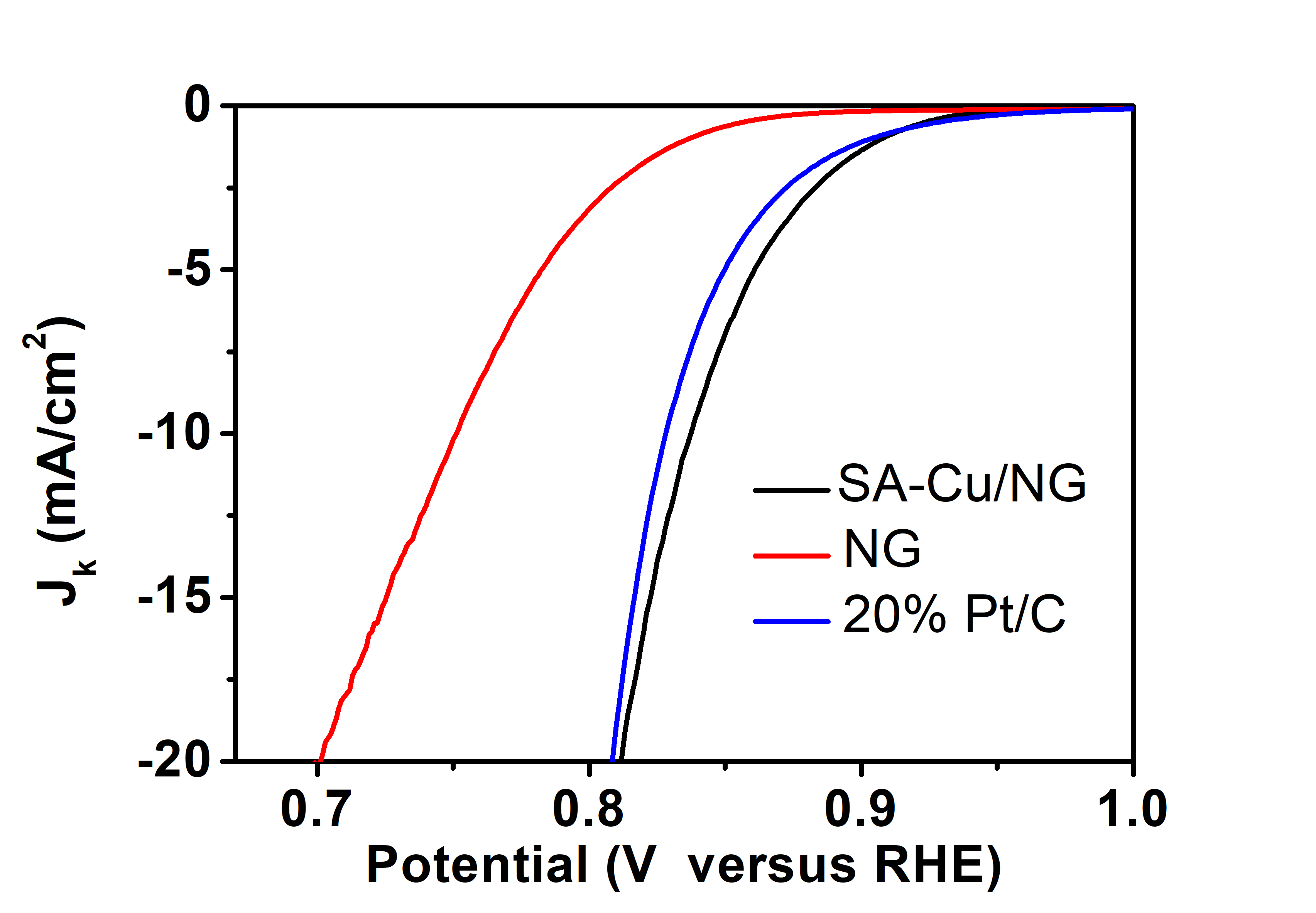
**

**Figure S9** J_k_ of SA-Cu/NG, NG and 20% Pt/C in alkaline media.

**
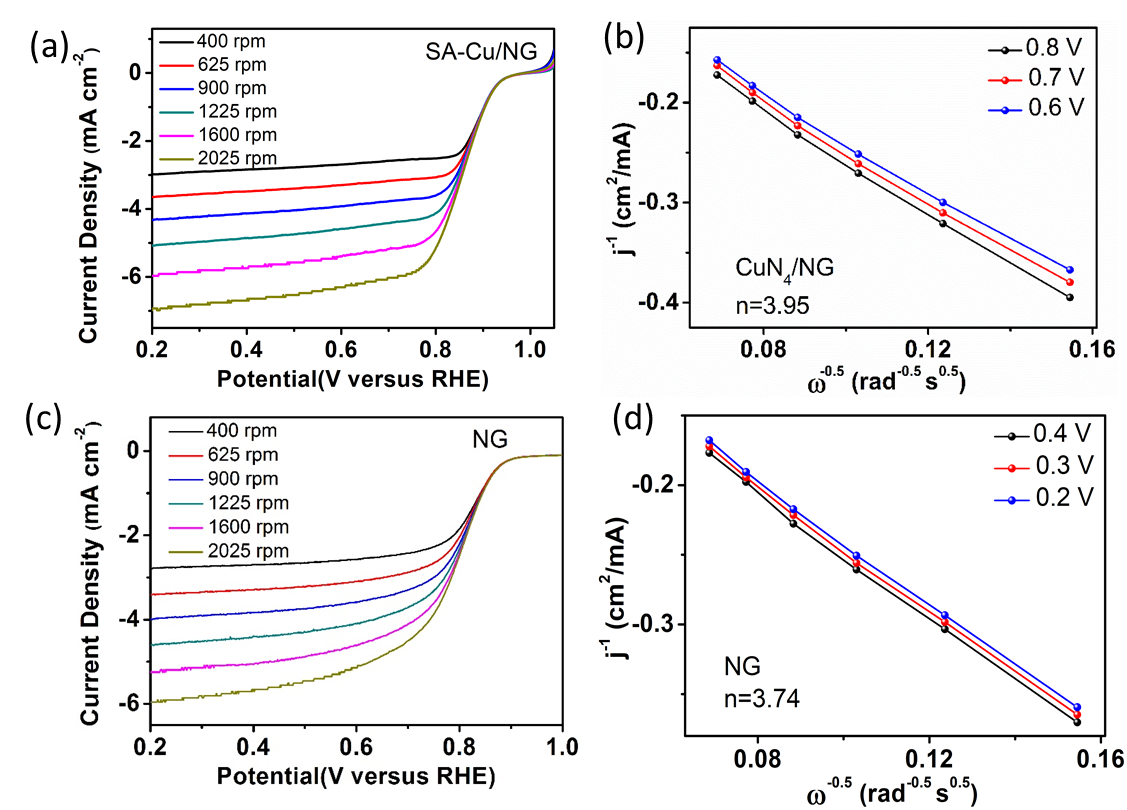
**

**Figure S10** LSV of (a) SA-Cu/NG, (c) NG under 0.1 M KOH condition. K-L curves of (b) SA-Cu/NG, (d) NG.

**
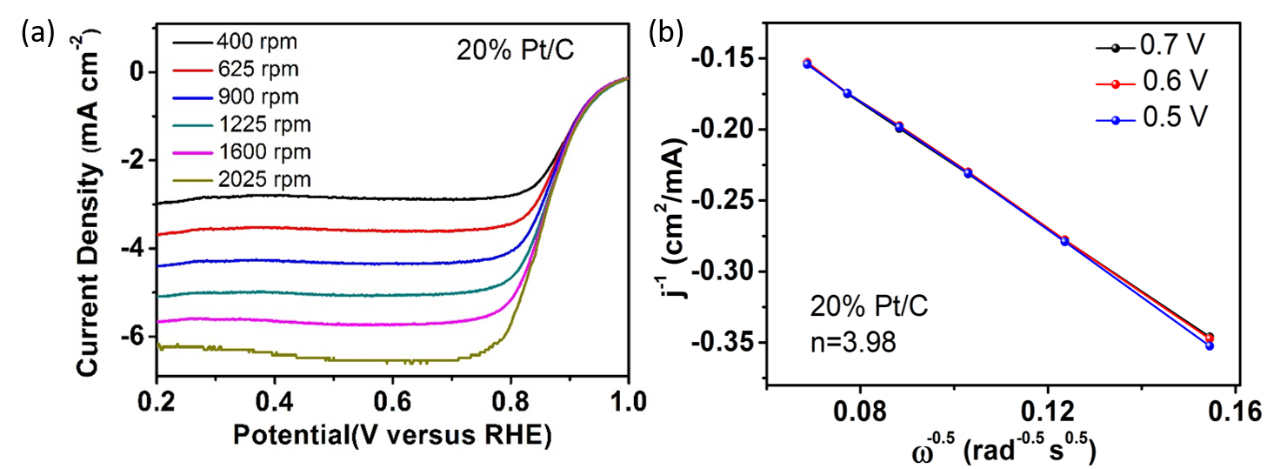
**

**Figure S11** (a) LSV and (b) K-L curves of 20 % Pt/C under 0.1 M KOH solution.

**
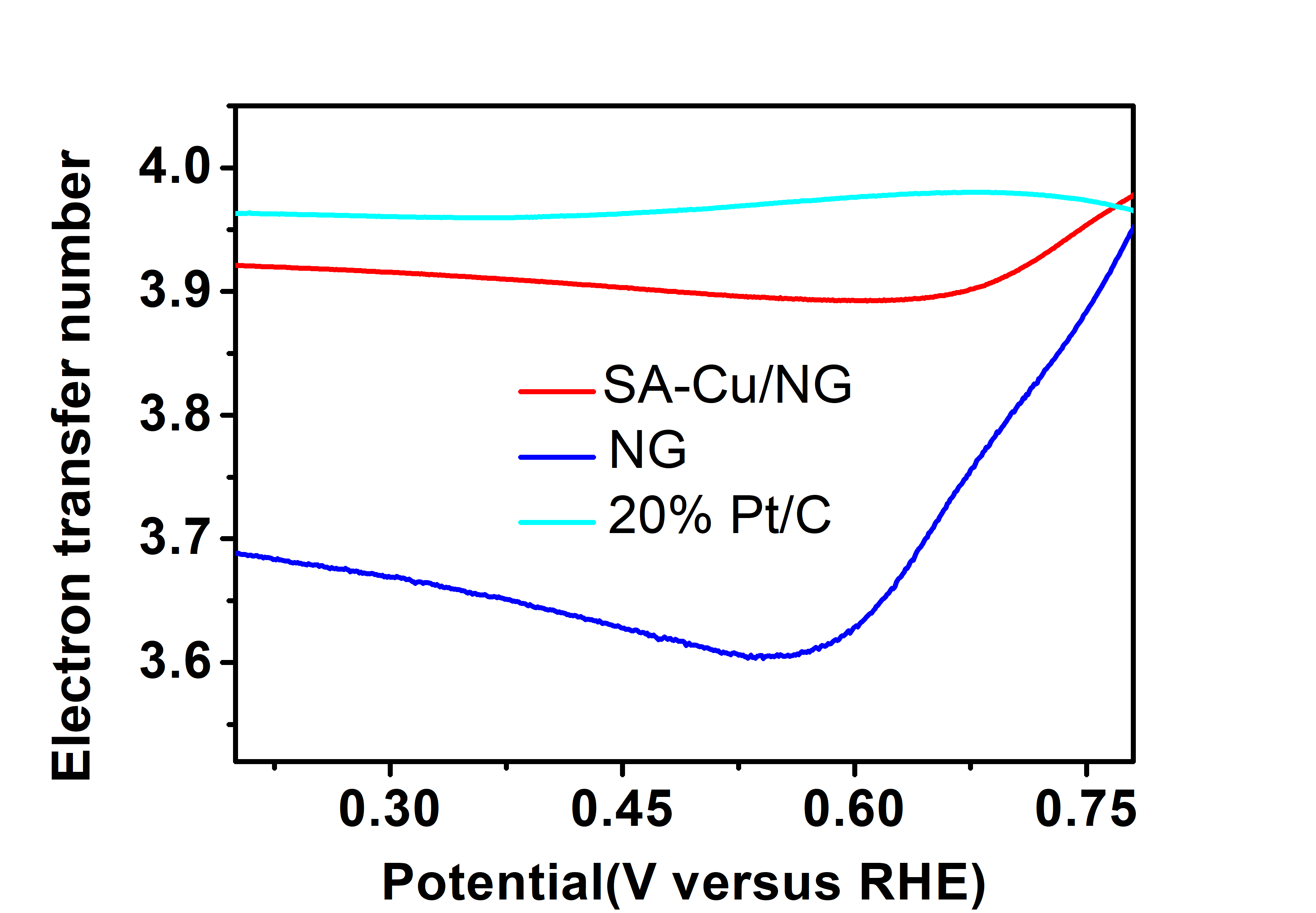
**

**Figure S12** The n from 0.2 V to 0.8 V for SA-Cu/NG, NG and 20 % Pt/C in alkaline media.

**
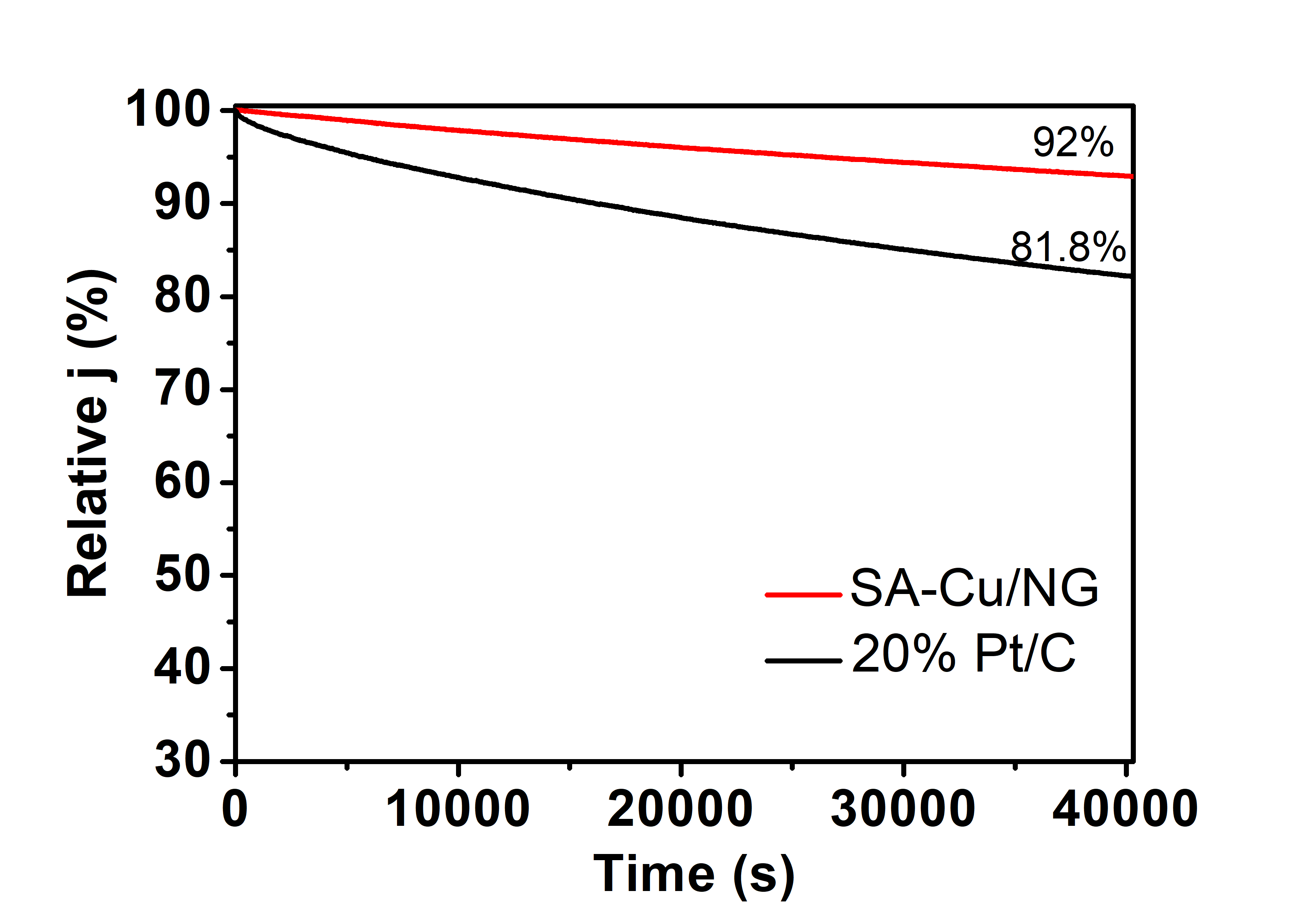
**

**Figure S13** i–t measurement of SA-Cu/NG and 20% Pt/C in alkaline media at 0.6 V (versus RHE).

**
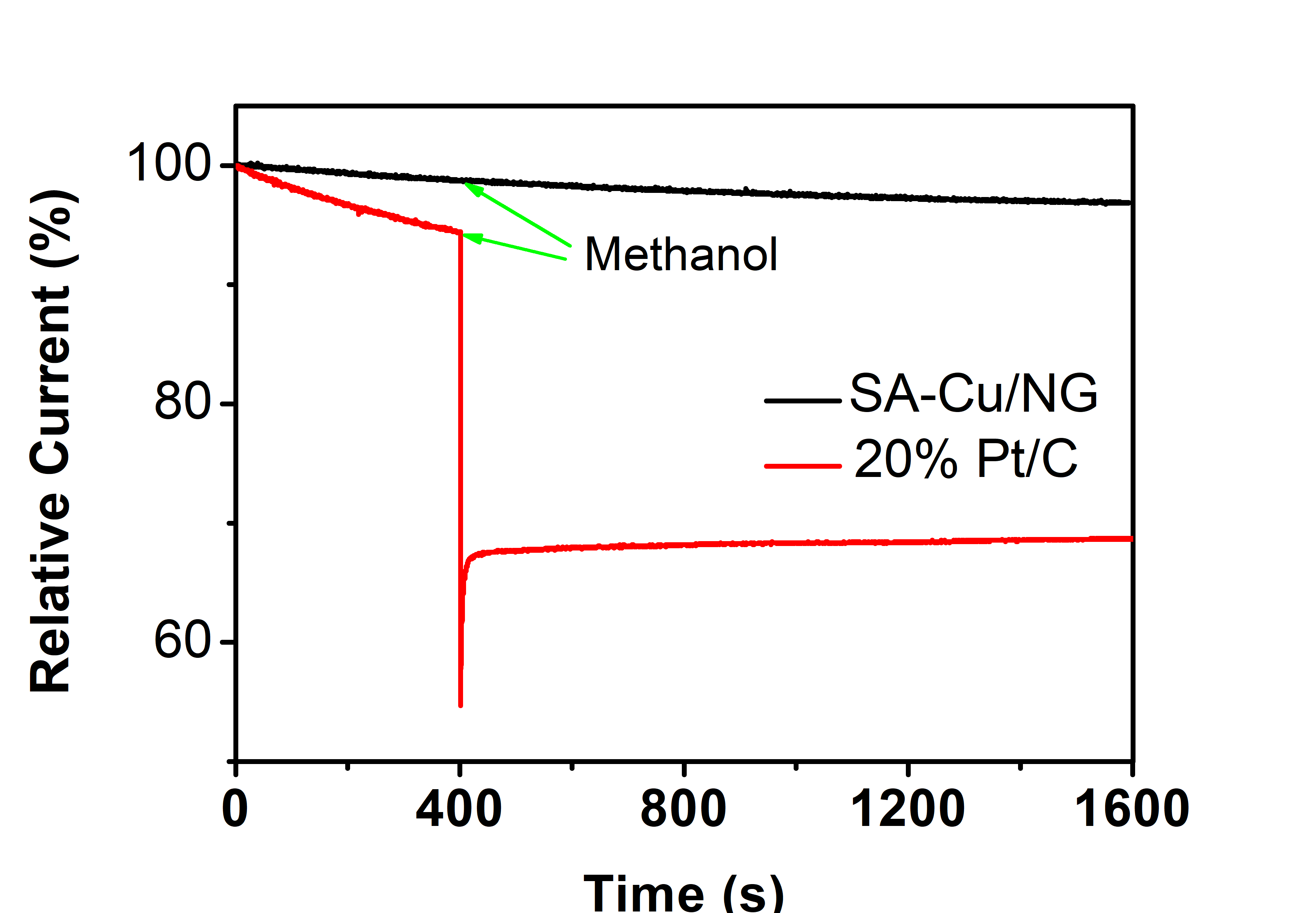
**

**Figure S14** I–t measurement of SA-Cu/NG and 20% Pt/C by dropping CH_3_OH after ~ 400 s.


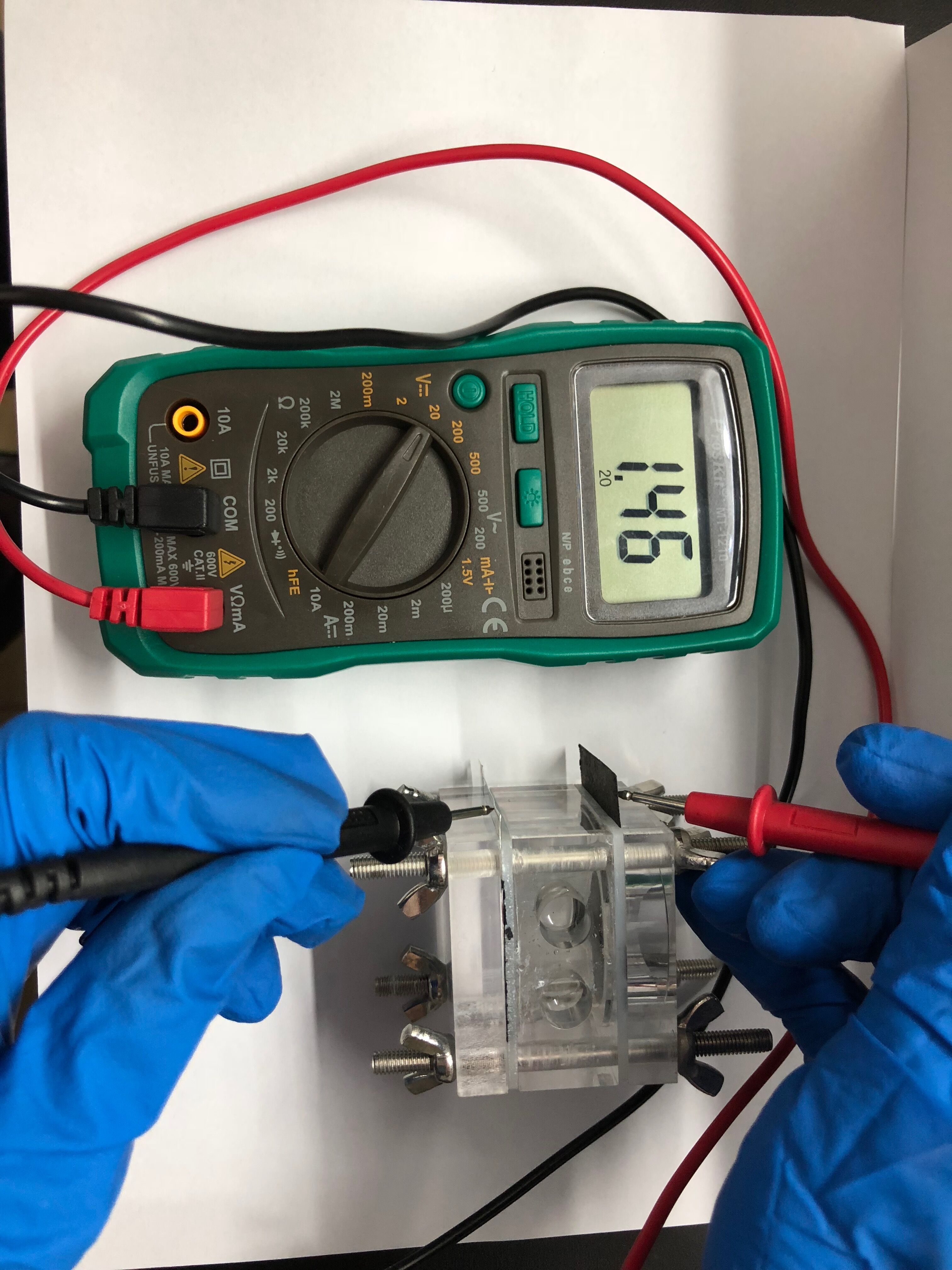


**Figure S15** Open circuit voltage of SA-Cu/NG assembled battery is measured by universal meter.

**
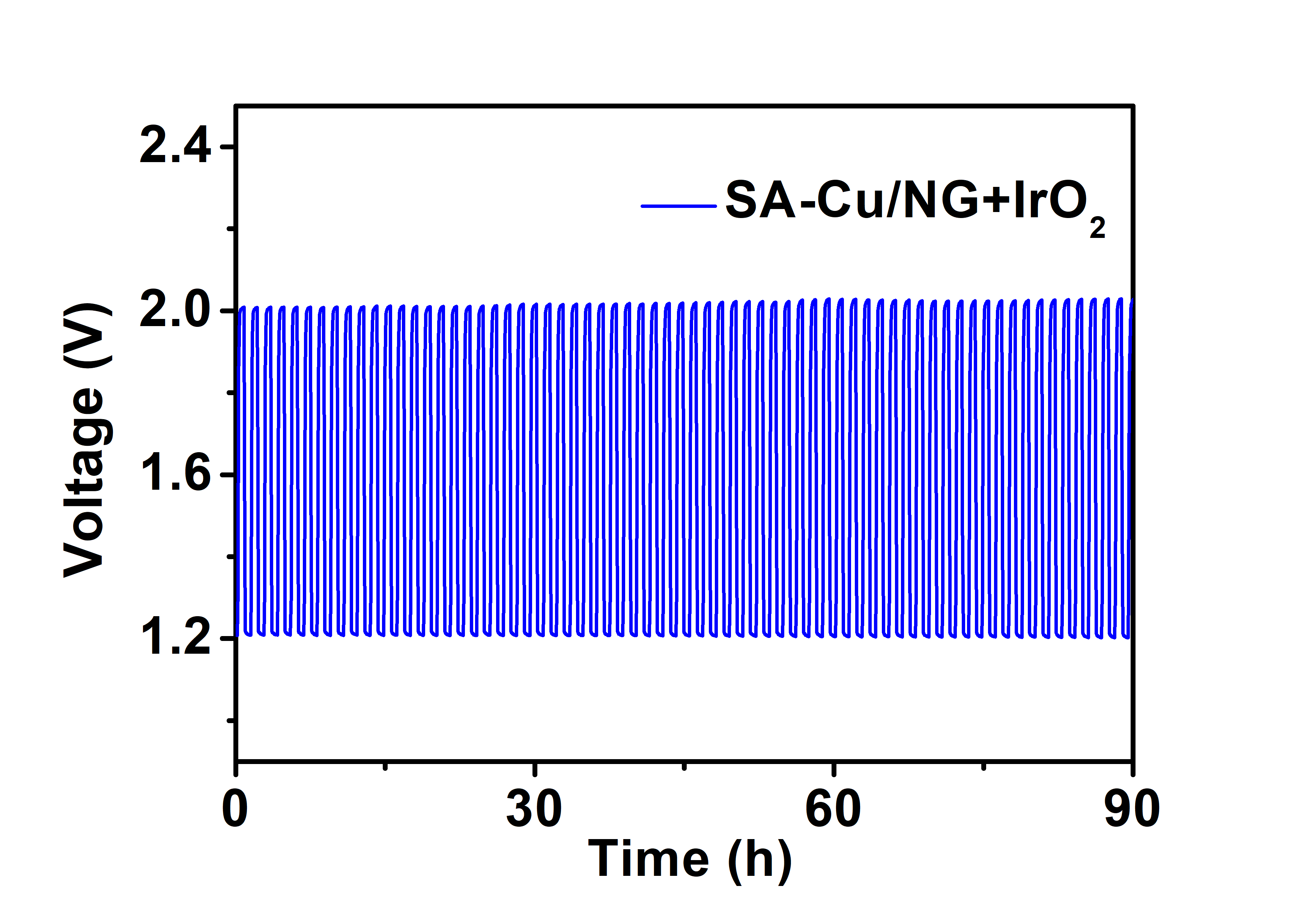
**

**Figure S16** The stability test for SA-Cu/NG+IrO_2_ based battery for 90 h.

**
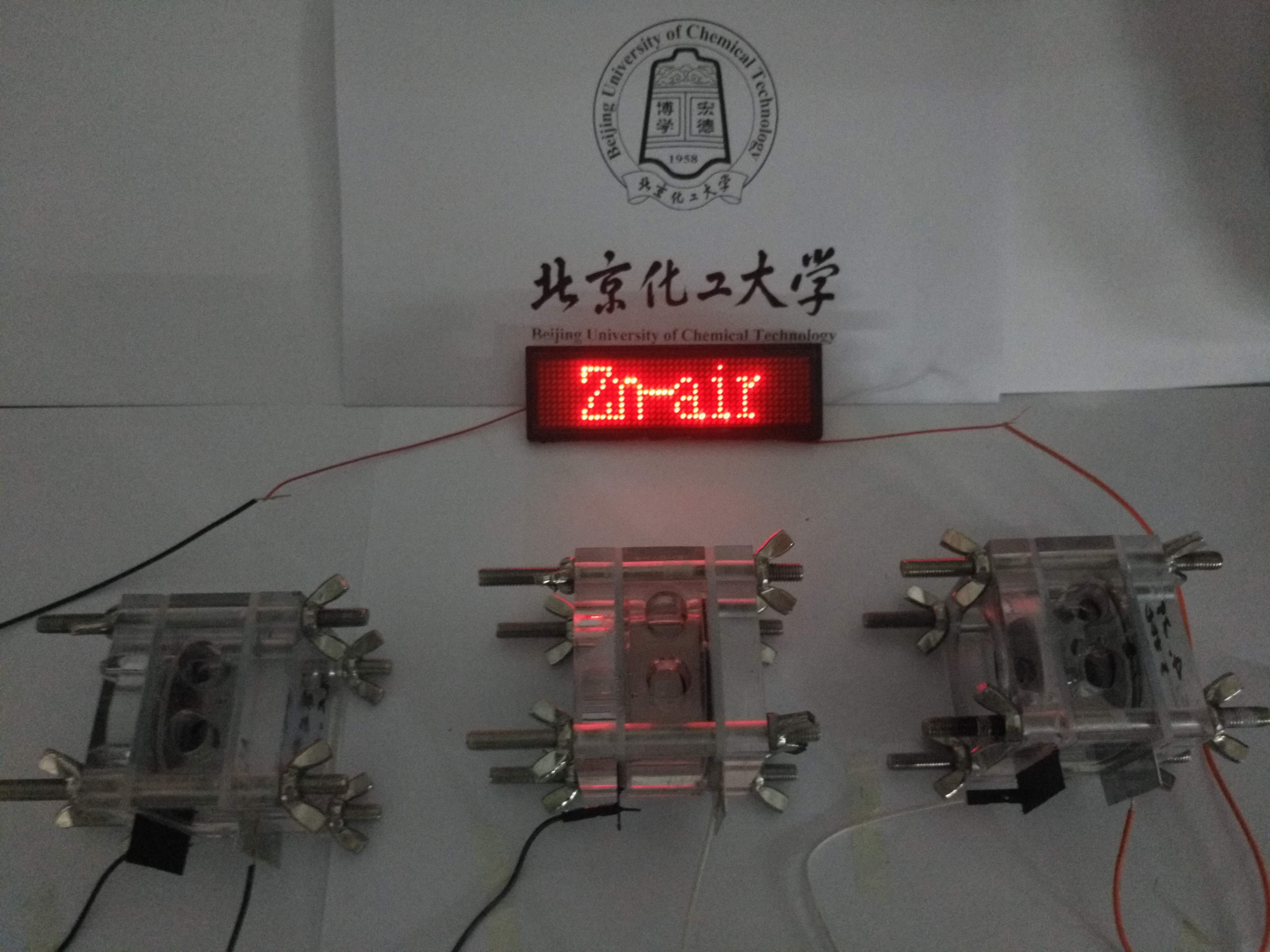
**

**Figure S17** The picture of LED lighted by three SA-Cu/NG+IrO_2_ based batteries.


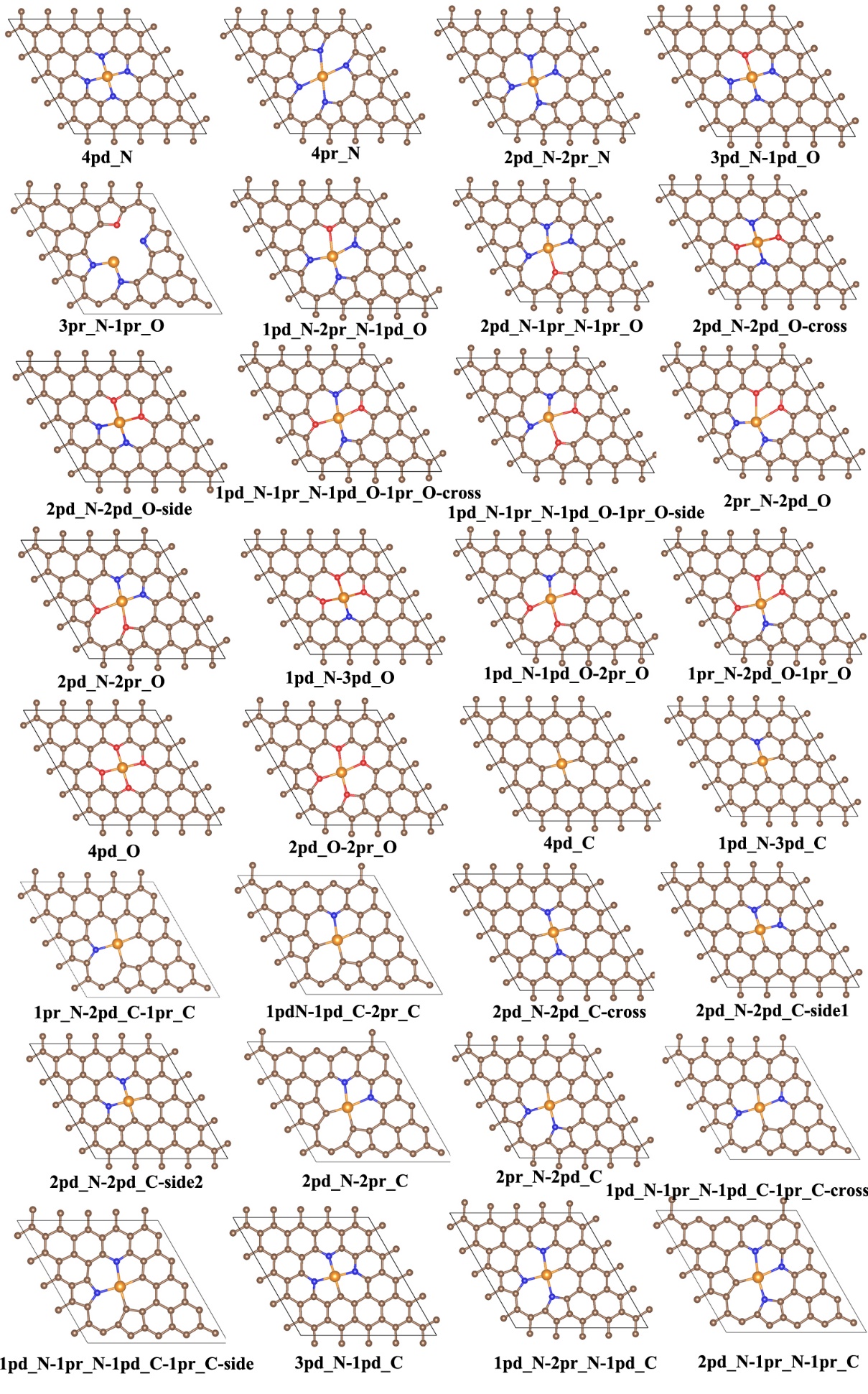


**Figure S18**. Top view of all possible structures of Cu SACs. “pd” means pyridine and “pr” means pyrrole.


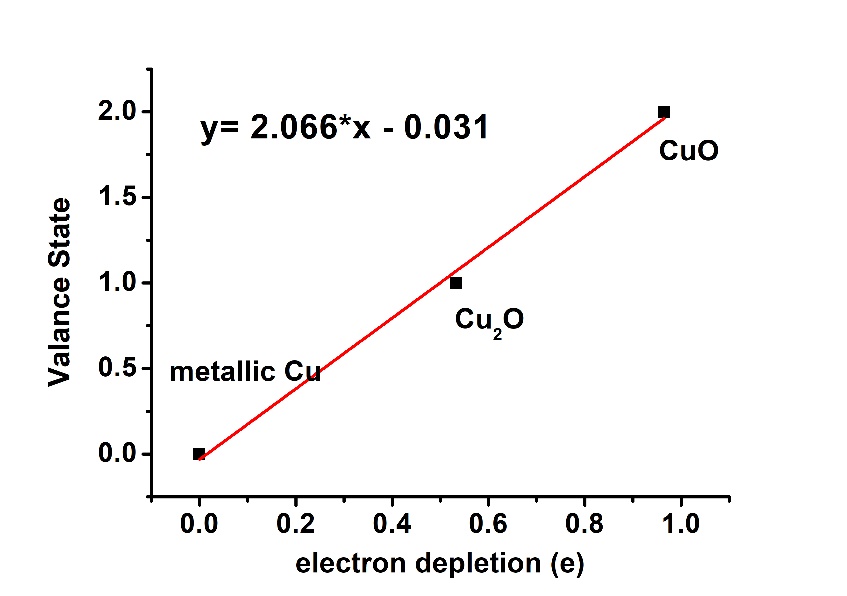


**Figure S19**. The scaling relationship between electron depletion of Cu atoms and valance state of Cu.


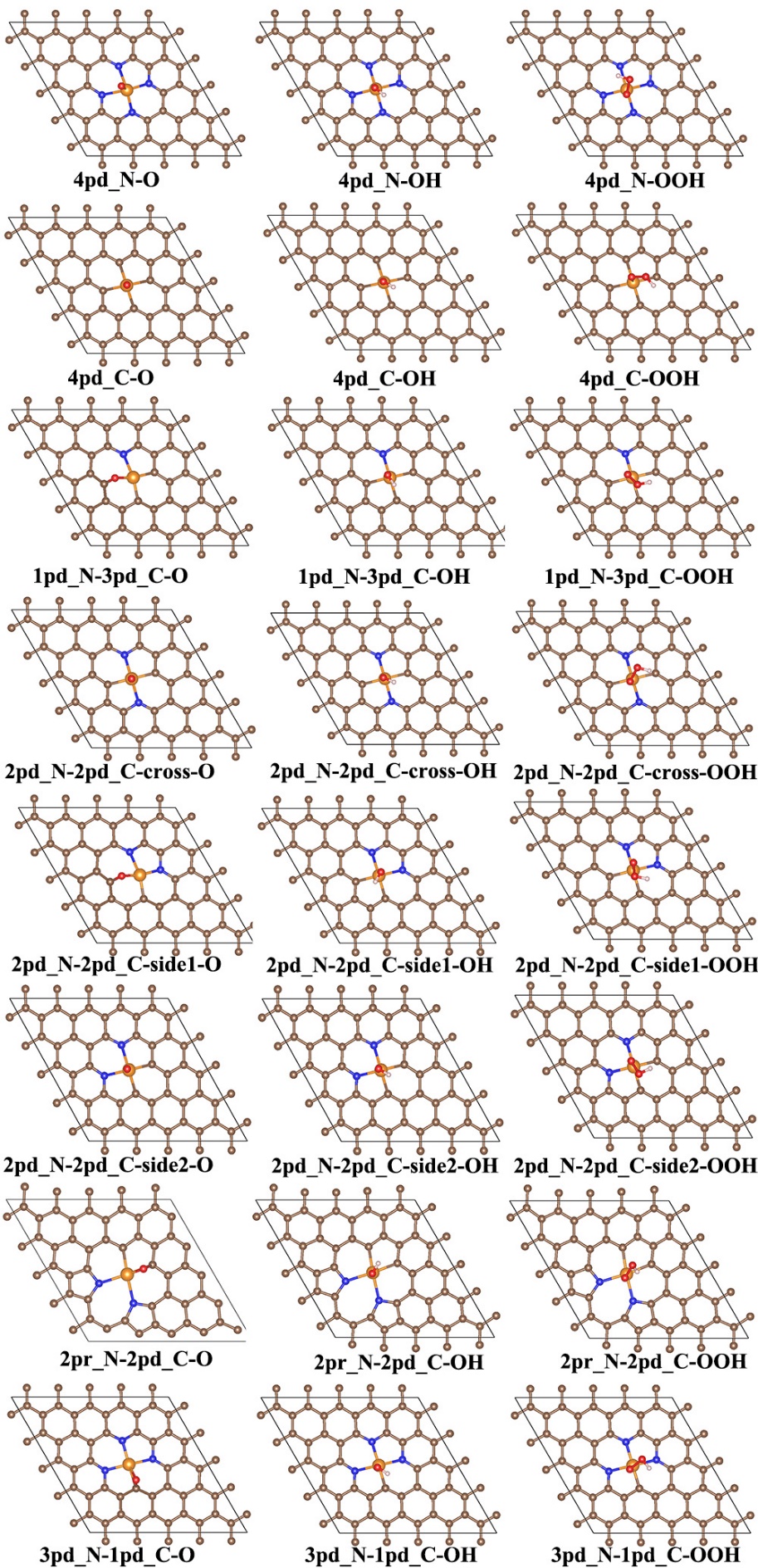


**Figure S20**. Top view of relaxed configurations of intermediate along ORR adsorbed on Cu SACs. “pd” means pyridine and “pr” means pyrrole.


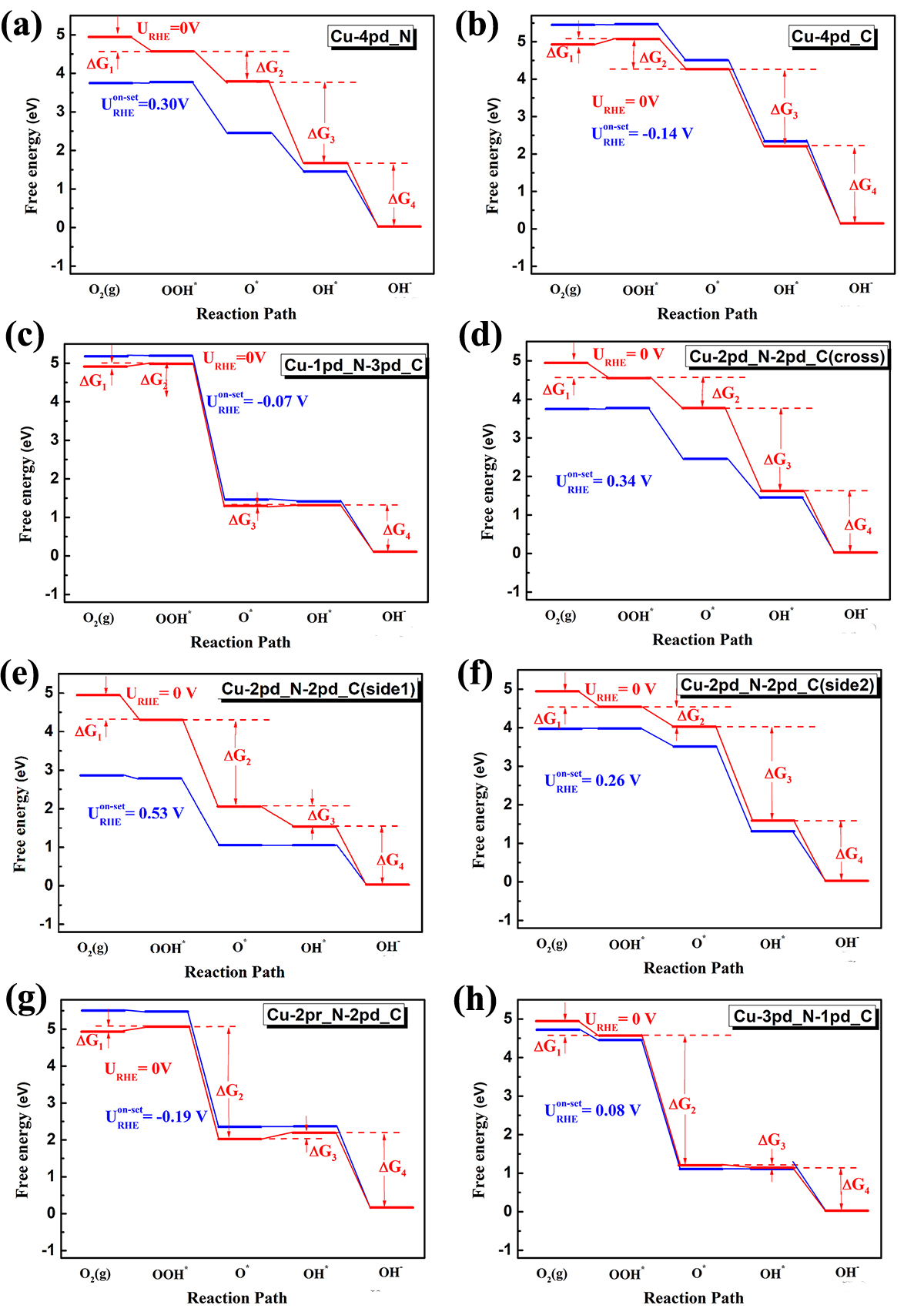


**Figure S21**. Free energy diagram for 4e^-^ transfer ORR on Cu single atom catalysts at zero electrode potential and on-set electrode potential with reversible hydrogen electrode (RHE) in an alkaline electrolyte. “pd” means pyridine and “pr” means pyrrole.


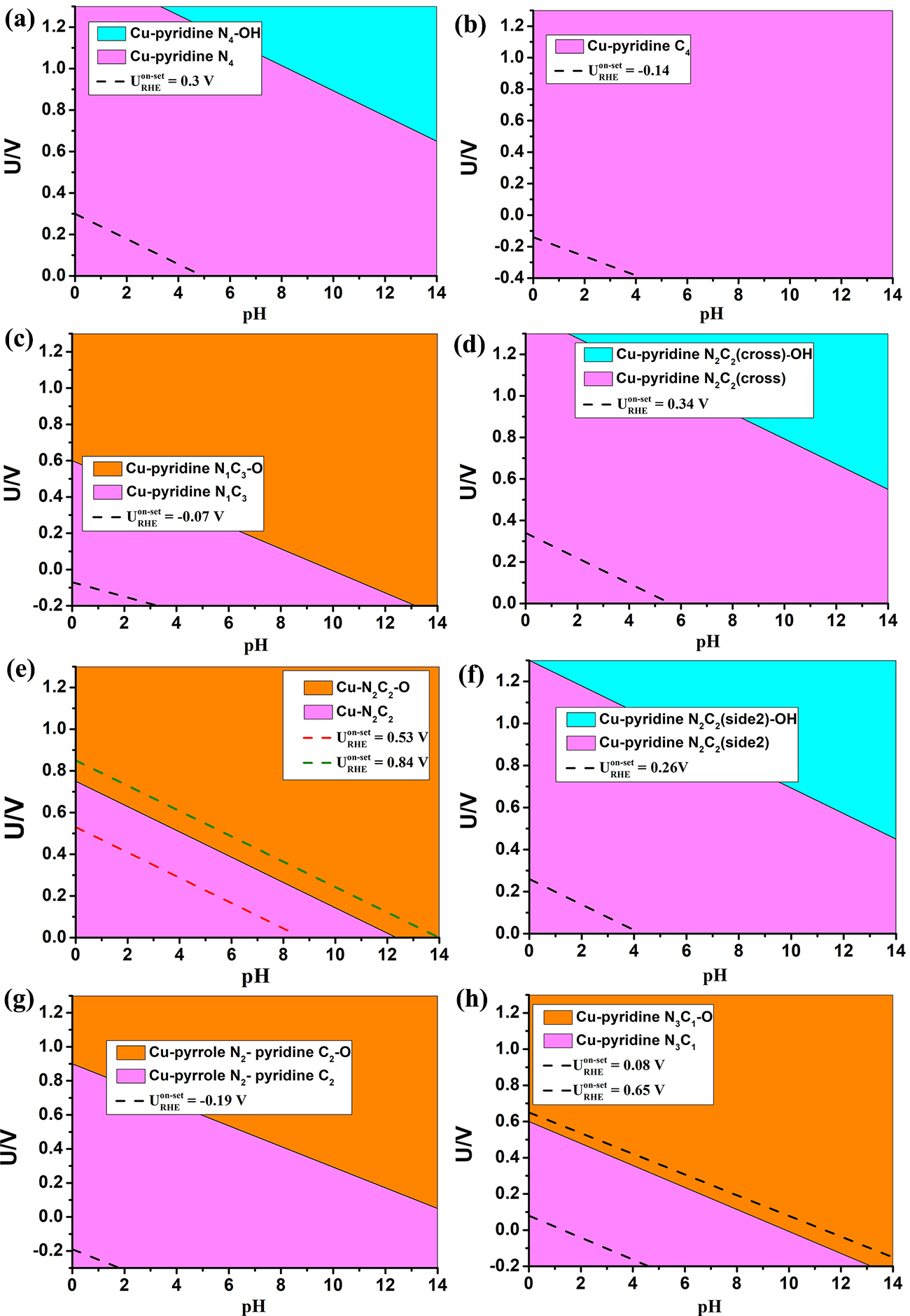


**Figure S22.** Surface Pourbaix diagrams of Cu SACs. The values of on-set potential are determined self-consistently.

**
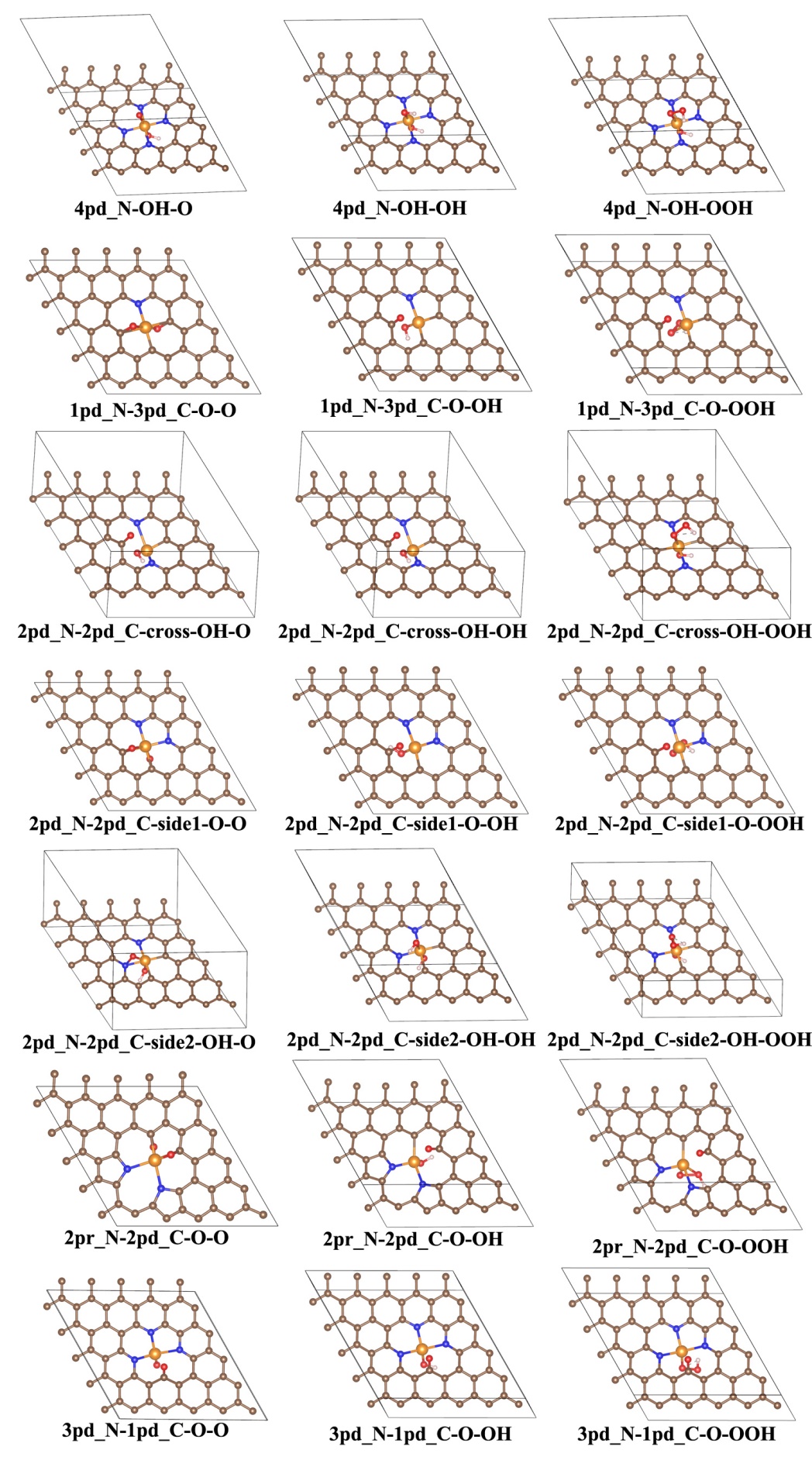
**

**Figure S23**. Top view of relaxed configurations of intermediate along ORR adsorbed on intermediate-modified Cu single atom catalysts. “pd” means pyridine and “pr” means pyrrole.


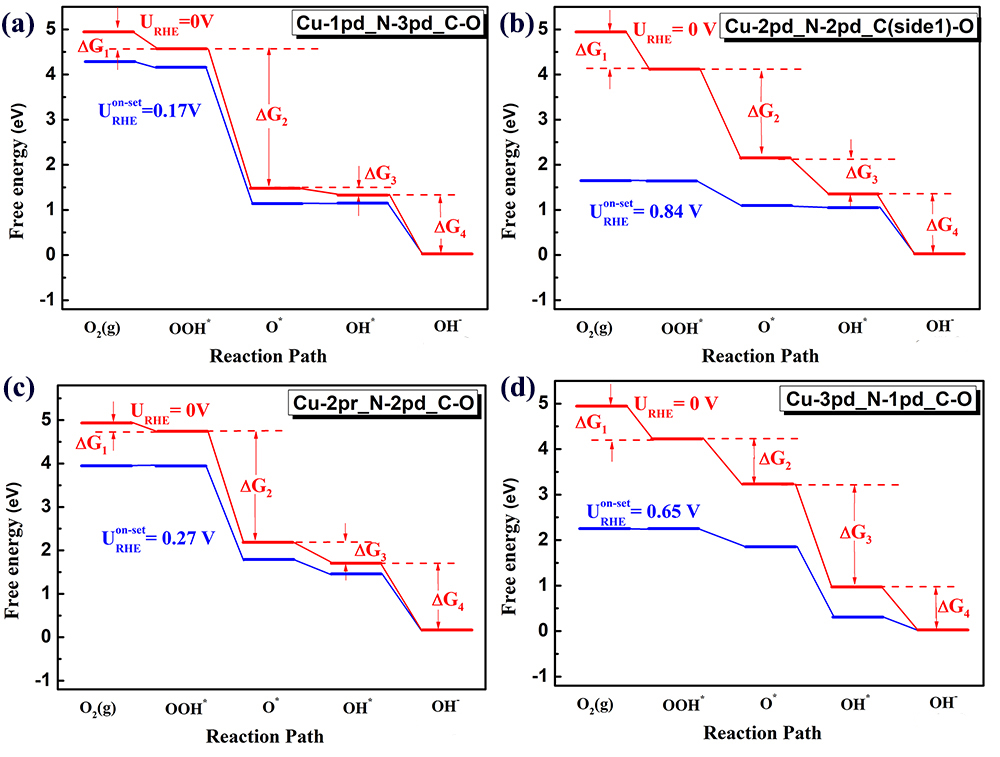


**Figure S24**. Free energy diagram for 4e^-^ transfer ORR on Cu single atom catalysts at zero electrode potential and on-set electrode potential with reversible hydrogen electrode (RHE) in an alkaline electrolyte. “pd” means pyridine and “pr” means pyrrole.

**
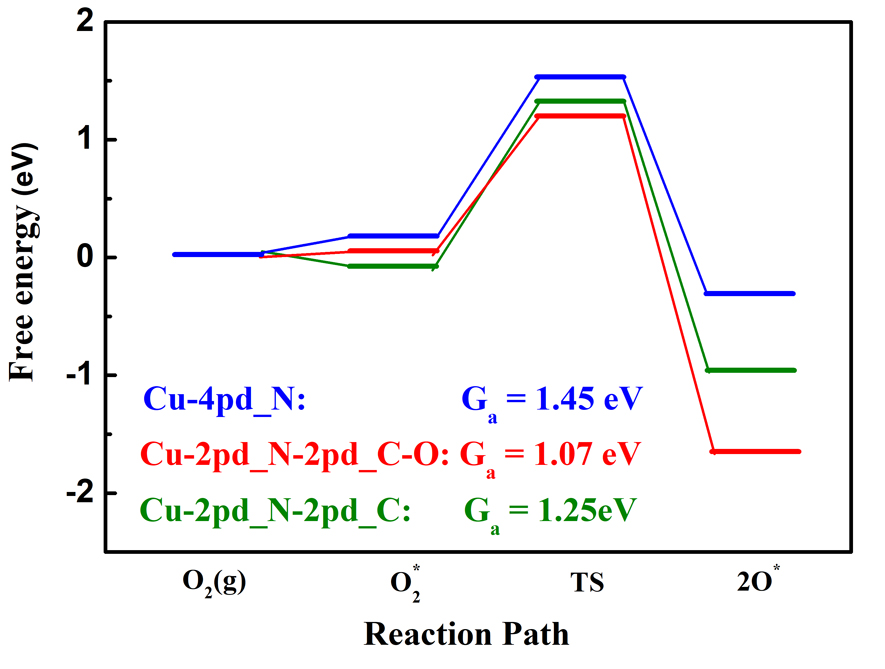
**

**Figure S25.** Free energy diagram for O_2_ dissociation pathway on Cu-4pd_N, Cu-2pd_N-2pd­­­_C-side1 and Cu-2pd_N-2pd­­­_C-side1-O to form two adsorbed O^*^. “pd” means pyridine and “pr” means pyrrole.

**
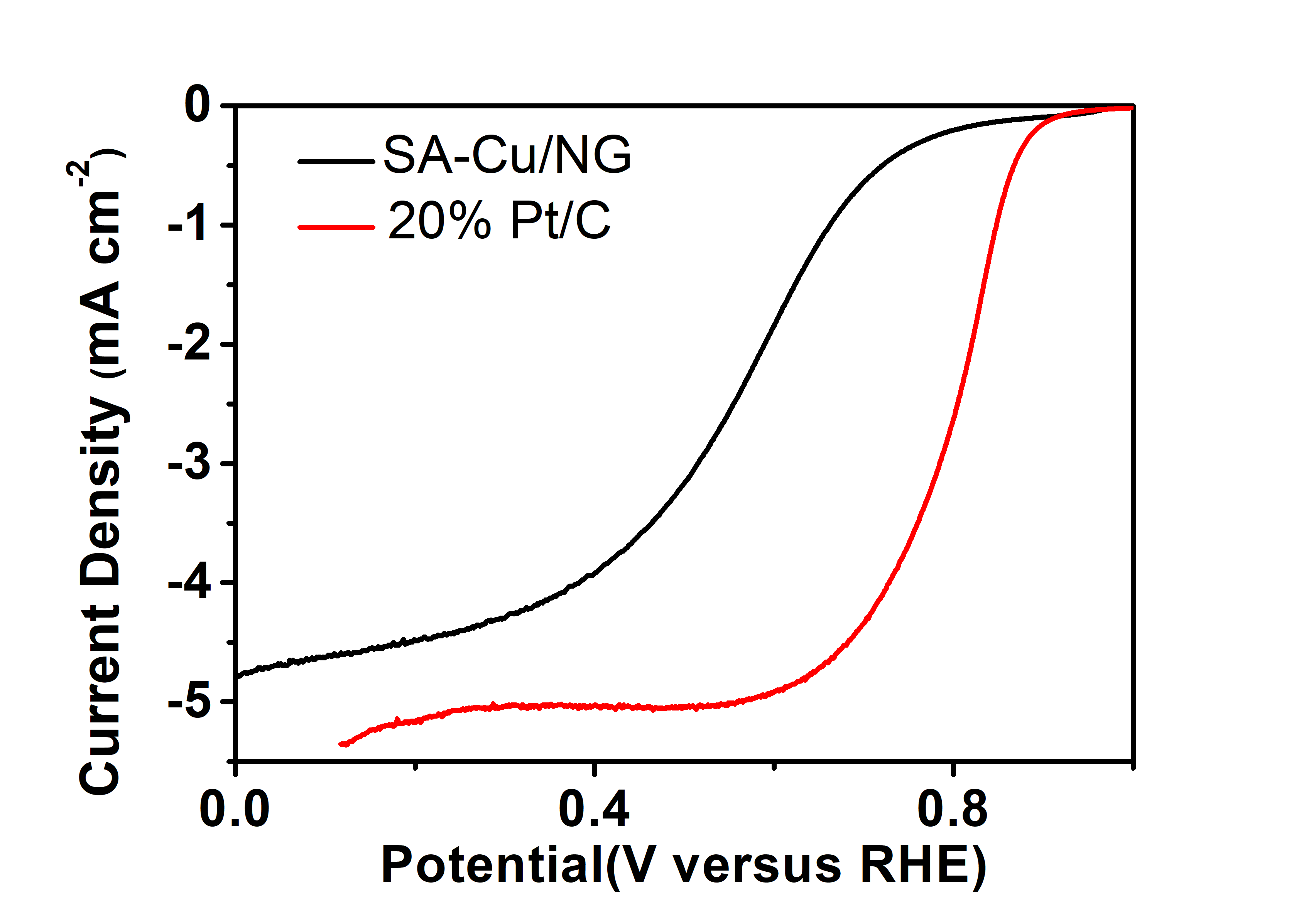
**

**Figure S26** (a) LSV curves of SA-Cu/NG and 20% Pt/C in 0.5 M H_2_SO_4_ media.

**Section S4: Supporting Tables**

**Table S1** Summary of XPS data of SA-Cu/NG and NG


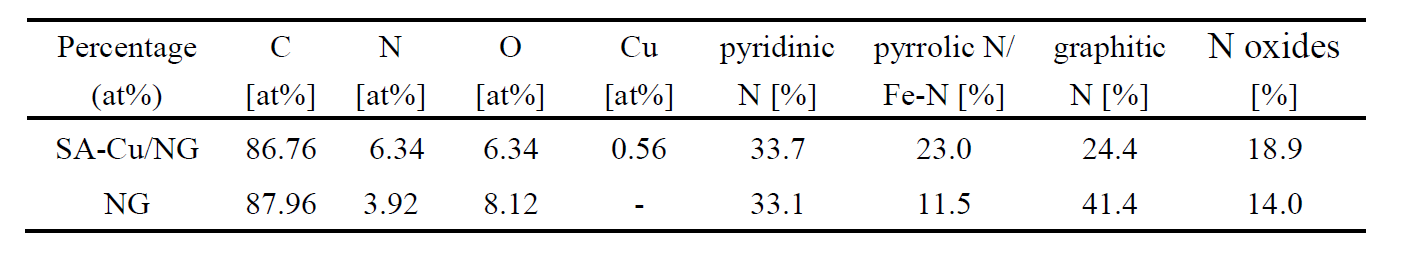


**Table S2** Parameters of EXAFS fits for SA-Cu/NG


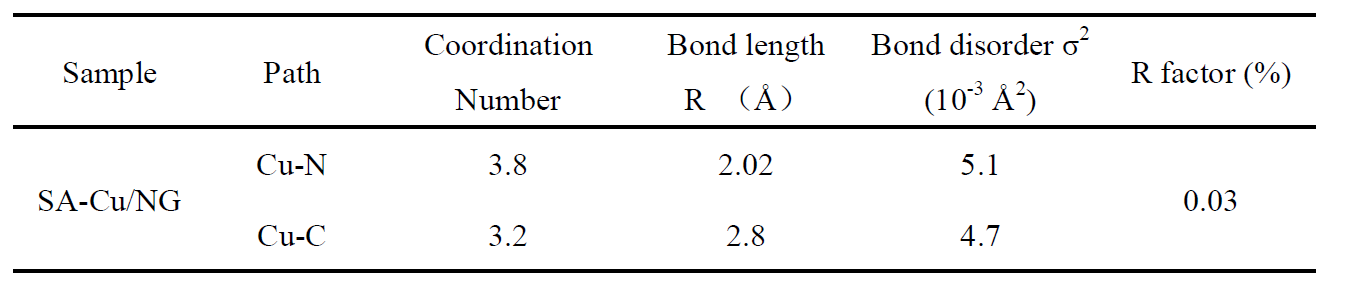


**Table S3** The porosity of SA-Cu/NG and NG


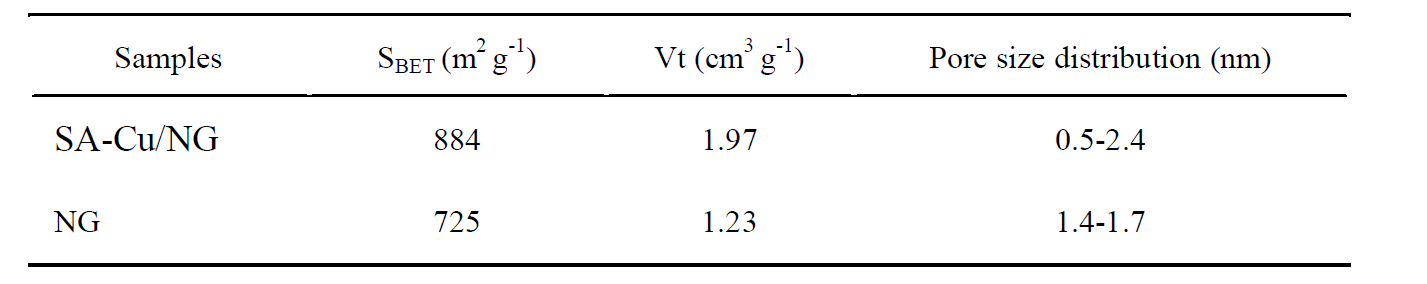


**Table S4.** The valance of Cu atom, Anti-aggregation Energy, Anti-leach Energy and Bond Length of Cu-neighbor atom for each possible active center of different Cu SACs shown in Figure S19.

| Coordination Environment of single Cu atom | Valance of Cu Atom | Anti-aggregation Energy (eV) | Anti-leach Energy (eV) | Bond Length of Cu-neighbor atom (Å) |
| --- | --- | --- | --- | --- |
| *4pd_N* | 1.94 | -1.55 | -0.86 | 1.92ⅹ4 |
| *4pr_N* | 1.69 | -0.61 | 0.08 | 2.41ⅹ4 |
| *2pd_N-2pr_N* | 1.86 | -2.31 | -1.63 | 2.06ⅹ2, 2.24ⅹ2 |
| *3pd_N-1pd_O* | 1.82 | -0.06 | 0.62 | 1.89ⅹ2, 1.93, 2.03 |
| *3pr_N-1pr_O* | 1.54 | -0.61 | 0.08 | 1.86ⅹ2, 3.22ⅹ2 |
| *1pd_N-2pr_N-1pd_O* | 1.66 | -1.29 | -0.61 | 2.03, 1.94, 2.27, 2.47 |
| *2pd_N-1pr_N-1pr_O* | 1.55 | -0.89 | -0.21 | 2.02, 2.03, 2.25, 2.41 |
| *2pd_N-2pd_O-cross* | 1.48 | 1.28 | 1.96 | 1.91ⅹ2, 2.08ⅹ2 |
| *2pd_N-2pd_O-side* | 1.58 | 1.54 | 2.22 | 1.92ⅹ2, 2.05ⅹ2 |
| *1pd_N-1pr_N-1pd_O-1pr_O-cross* | 1.42 | 0.26 | 0.94 | 2.16, 2.04, 2.20, 2.28 |
| *1pd_N-1pr_N-1pd_O-1pr_O-side* | 1.30 | 0.07 | 0.75 | 1.91ⅹ2, 2.52ⅹ2 |
| *2pr_N-2pd_O* | 1.41 | -0.38 | 0.30 | 1.84ⅹ2, 2.67ⅹ2 |
| *2pd_N-2pr_O* | 1.40 | 0.65 | 1.33 | 2.20ⅹ2, 2.36ⅹ2 |
| *1pd_N-3pd_O* | 1.47 | 2.23 | 2.91 | 1.90, 2.05, 2.10ⅹ2 |
| *1pd_N-1pd_O-2pr_O* | 1.45 | 1.35 | 2.03 | 1.94, 2.12, 2.29, 2.40 |
| *1pr_N-2pd_O-1pr_O* | 1.40 | 1.09 | 1.77 | 1.90, 2.26, 2.42, 2.20 |
| *4pd_O* | 1.56 | 3.93 | 4.61 | 1.95ⅹ4 |
| *2pd_O2pr_O* | 1.54 | 2.87 | 3.55 | 2.14ⅹ2, 2.21ⅹ2 |
| *4pd_C* | 1.43 | -1.32 | -0.63 | 1.89ⅹ4 |
| *1pd_N-3pd_C* | 1.54 | -3.05 | -2.36 | 1.97, 1.95ⅹ2, 1.94 |
| *1pr_N-2pd_C-1pr_C* | 1.52 | -4.43 | -3.75 | 2.05, 2.00ⅹ2, 2.22 |
| *1pd_N-1pd_C-2pr_C* | 1.49 | -3.94 | -3.26 | 2.24, 2.12, 2.01, 1.99 |
| *2pd_N-2pd_C-cross* | 1.75 | -2.85 | -2.17 | 1.96ⅹ2, 1.95ⅹ2 |
| *2pd_N-2pd_C-side1* | 1.65 | -3.24 | -2.56 | 1.99ⅹ2, 1.98ⅹ2 |
| *2pd_N-2pd_C-side2* | 1.62 | -2.38 | -1.69 | 1.98ⅹ2, 1.96ⅹ2 |
| *2pd_N-2pr_C* | 1.61 | -3.97 | -3.28 | 1.99ⅹ2, 2.24ⅹ2 |
| *2pr_N-2pd_C* | 1.53 | -2.45 | -1.77 | 2.10ⅹ2, 2.12ⅹ2 |
| *1pd_N-1pr_N-1pd_C-1pr_C-cross* | 1.69 | -3.00 | -2.31 | 2.04,2.05,2.12,2.23 |
| *1pd_N-1pr_N-1pd_C-1pr_C-side* | 1.60 | -3.48 | -2.79 | 2.04,2.07,2.12,2,23 |
| *3pd_N-1pd_C* | 1.80 | -2.22 | -1.54 | 1.91, 1.89ⅹ2, 1.92 |
| *1pd_N-2pr_N-1pd_C* | 1.74 | -2.83 | -2.15 | 2.08, 2.09, 2.12, 2.23 |
| *2pd_N-1pr_N-1pr_C* | 1.70 | -2.51 | -1.83 | 1.98, 2.03, 2.27, 2.28 |

**Table S5.** Values used for the corrections in determining the free energy of free H_2_ and H_2_O molecule.

| **Species** | **T×S (eV) (298K)** | **ZPE (eV)** |
| --- | --- | --- |
| H_2_ | 0.41 | 0.27 |
| H_2_O(0.035 bar) | 0.67 | 0.56 |

**Table S6.** DFT adsorption energies (ΔE_ad_, eV), zero-point energy (ZPE, eV), zero-point energy correction (ΔZPE, eV), entropy (T*S, eV, T=298K), entropy correction (T*ΔS, eV, T=298K), adsorption free energies (ΔG_ad_, eV) of the adsorbed O along reaction pathway of ORR on different Cu SACs. Adsorption energies (eV) were taken relative to energies of free H_2_ and H_2_O molecule.

|  | **ΔE_ad_O** | **ZPE_O_** | **ΔZPE_O_** | **T*S_O_** | **T*ΔS_O_** | **ΔG_ad_O** |
| --- | --- | --- | --- | --- | --- | --- |
| *4pd_N* | 4.02 | 0.05 | -0.24 | 0.11 | -0.15 | 3.93 |
| *4pd_C* | 4.26 | 0.04 | -0.25 | 0.17 | -0.09 | 4.10 |
| *1pd_N-3pd_C* | 1.32 | 0.11 | -0.18 | 0.03 | -0.23 | 1.37 |
| *2pd_N-2pd_C-cross* | 3.97 | 0.03 | -0.26 | 0.06 | -0.20 | 3.91 |
| *2pd_N-2pd_C-side1* | 1.91 | 0.11 | -0.18 | 0.03 | -0.23 | 1.97 |
| *2pd_N-2pd_C-side2* | 4.13 | 0.03 | -0.26 | 0.12 | -0.14 | 4.01 |
| *2pr_N-2pd_C* | 1.93 | 0.11 | -0.18 | 0.03 | -0.23 | 1.98 |
| *3pd_N-1pd_C* | 1.30 | 0.09 | -0.20 | 0.04 | -0.22 | 1.32 |

**Table S7.** DFT adsorption energies (ΔE_ad_, eV), zero-point energy (ZPE, eV), zero-point energy correction (ΔZPE, eV), entropy (T*S, eV, T=298K), entropy correction (T*ΔS, eV, T=298K), adsorption free energies (ΔG_ad_, eV) of the adsorbed OH along reaction pathway of ORR on different Cu SACs. Adsorption energies (eV) were taken relative to energies of free H_2_ and H_2_O molecule.

|  | **ΔE_ad_OH** | **ZPE_OH_** | **ΔZPE_OH_** | **T*S_OH_** | **T*ΔS_OH_** | **ΔG_ad_OH** |
| --- | --- | --- | --- | --- | --- | --- |
| *4pd_N* | 1.54 | 0.32 | -0.11 | 0.17 | -0.30 | 1.73 |
| *4pd_C* | 1.87 | 0.33 | -0.09 | 0.15 | -0.31 | 2.09 |
| *1pd_N-3pd_C* | 1.23 | 0.31 | -0.12 | 0.19 | -0.28 | 1.40 |
| *2pd_N-2pd_C-cross* | 1.46 | 0.31 | -0.11 | 0.18 | -0.29 | 1.63 |
| *2pd_N-2pd_C-side1* | 1.24 | 0.31 | -0.11 | 0.16 | -0.31 | 1.44 |
| *2pd_N-2pd_C-side2* | 1.36 | 0.32 | -0.10 | 0.14 | -0.33 | 1.59 |
| *2pr_N-2pd_C* | 1.94 | 0.32 | -0.10 | 0.14 | -0.33 | 2.17 |
| *3pd_N-1pd_C* | 1.05 | 0.31 | -0.11 | 0.16 | -0.30 | 1.24 |

**Table S8.** DFT adsorption energies (ΔE_ad_, eV), zero-point energy (ZPE, eV), zero-point energy correction (ΔZPE, eV), entropy (T*S, eV, T=298K), entropy correction (T*ΔS, eV, T=298K), adsorption free energies (ΔG_ad_, eV) of the adsorbed OOH along reaction pathway of ORR on different Cu SACs. Adsorption energies (eV) were taken relative to energies of free H_2_ and H_2_O molecule.

|  | **ΔE_ad_OOH** | **ZPE_OOH_** | **ΔZPE_OOH_** | **T*S_OOH_** | **T*ΔS_OOH_** | **ΔG_ad_OOH** |
| --- | --- | --- | --- | --- | --- | --- |
| *4pd_N* | 4.46 | 0.42 | -0.30 | 0.27 | -0.46 | 4.62 |
| *4pd_C* | 4.82 | 0.42 | -0.30 | 0.19 | -0.53 | 5.06 |
| *1pd_N-3pd_C* | 4.72 | 0.38 | -0.34 | 0.11 | -0.61 | 4.99 |
| *2pd_N-2pd_C-cross* | 4.38 | 0.43 | -0.29 | 0.24 | -0.49 | 4.58 |
| *2pd_N-2pd_C-side1* | 4.14 | 0.42 | -0.30 | 0.28 | -0.45 | 4.29 |
| *2pd_N-2pd_C-side2* | 4.42 | 0.41 | -0.30 | 0.19 | -0.54 | 4.66 |
| *2pr_N-2pd_C* | 4.82 | 0.40 | -0.32 | 0.18 | -0.55 | 5.05 |
| *3pd_N-1pd_C* | 4.44 | 0.42 | -0.30 | 0.28 | -0.45 | 4.59 |

**Table S9.** Reaction free energy (in eV vs RHE) of elementary step at U_RHE_=0V and onset potential (in V vs RHE) for ORR on different Cu SACs.

|  | 𝛥𝐺_1_ | 𝛥𝐺_2_ | 𝛥𝐺_3_ | 𝛥𝐺_4_ | U_onset_ |
| --- | --- | --- | --- | --- | --- |
| *4pd_N* | -0.30 | -0.69 | -2.20 | -1.73 | 0.30 |
| *4pd_C* | 0.14 | -0.96 | -2.01 | -2.09 | -0.14 |
| *1pd_N-3pd_C* | 0.07 | -3.72 | 0.03 | -1.40 | -0.07 |
| *2pd_N-2pd_C-cross* | -0.34 | -0.66 | -2.28 | -1.63 | 0.34 |
| *2pd_N-2pd_C-side1* | -0.63 | -2.33 | -0.53 | -1.54 | 0.53 |
| *2pd_N-2pd_C-side2* | -0.26 | -0.65 | -2.42 | -1.59 | 0.26 |
| *2pr_N-2pd_C* | 0.13 | -3.07 | 0.19 | -2.17 | -0.19 |
| *3pd_N-1pd_C* | -0.33 | -3.27 | -0.08 | -1.24 | 0.08 |

**Table S10.** DFT adsorption energies (ΔE_ad_, eV), zero-point energy (ZPE, eV), zero-point energy correction (ΔZPE, eV), entropy (T*S, eV, T=298K), entropy correction (T*ΔS, eV, T=298K), adsorption free energies (ΔG_ad_, eV) of the adsorbed O along reaction pathway of ORR on O-reconstituted Cu SACs. Adsorption energies (eV) were taken relative to energies of free H_2_ and H_2_O molecule.

|  | **ΔE_ad_O** | **ZPE_O_** | **ΔZPE_O_** | **T*S_O_** | **T*ΔS_O_** | **ΔG_ad_O** |
| --- | --- | --- | --- | --- | --- | --- |
| *1pd_N-3pd_C-O* | 1.46 | 0.11 | -0.18 | 0.03 | -0.23 | 1.51 |
| *2pd_N-2pd_C-side1-O* | 2.04 | 0.10 | -0.19 | 0.03 | -0.23 | 2.08 |
| *2pr_N-2pd_C-O* | 2.06 | 0.11 | -0.18 | 0.04 | -0.22 | 2.10 |
| *3pd_N-1pd_C-O* | 3.41 | 0.05 | -0.24 | 0.09 | -0.17 | 3.34 |

**Table S11.** DFT adsorption energies (ΔE_ad_, eV), zero-point energy (ZPE, eV), zero-point energy correction (ΔZPE, eV), entropy (T*S, eV, T=298K), entropy correction (T*ΔS, eV, T=298K), adsorption free energies (ΔG_ad_, eV) of the adsorbed OH along reaction pathway of ORR on O-reconstituted Cu SACs. Adsorption energies (eV) were taken relative to energies of free H_2_ and H_2_O molecule.

|  | **ΔE_ad_OH** | **ZPE_OH_** | **ΔZPE_OH_** | **T*S_OH_** | **T*ΔS_OH_** | **ΔG_ad_OH** |
| --- | --- | --- | --- | --- | --- | --- |
| *1pd_N-3pd_C-O* | 1.05 | 0.35 | -0.07 | 0.10 | -0.36 | 1.33 |
| *2pd_N-2pd_C-side1-O* | 0.88 | 0.35 | -0.07 | 0.10 | -0.37 | 1.17 |
| *2pr_N-2pd_C-O* | 1.48 | 0.33 | -0.09 | 0.15 | -0.32 | 1.71 |
| *3pd_N-1pd_C-O* | 0.68 | 0.34 | -0.08 | 0.12 | -0.35 | 0.94 |

**Table S12.** DFT adsorption energies (ΔE_ad_, eV), zero-point energy (ZPE, eV), zero-point energy correction (ΔZPE, eV), entropy (T*S, eV, T=298K), entropy correction (T*ΔS, eV, T=298K), adsorption free energies (ΔG_ad_, eV) of the adsorbed OOH along reaction pathway of ORR on O-reconstituted Cu SACs. Adsorption energies (eV) were taken relative to energies of free H_2_ and H_2_O molecule.

|  | **ΔE_ad_OOH** | **ZPE_OOH_** | **ΔZPE_OOH_** | **T*S_OOH_** | **T*ΔS_OOH_** | **ΔG_ad_OOH** |
| --- | --- | --- | --- | --- | --- | --- |
| *1pd_N-3pd_C-O* | 4.26 | 0.44 | -0.28 | 0.14 | -0.58 | 4.57 |
| *2pd_N-2pd_C-side1-O* | 3.81 | 0.43 | -0.28 | 0.17 | -0.56 | 4.08 |
| *2pr_N-2pd_C-O* | 4.39 | 0.41 | -0.31 | 0.16 | -0.57 | 4.65 |
| *3pd_N-1pd_C-O* | 3.98 | 0.43 | -0.28 | 0.15 | -0.57 | 4.27 |

**Table S13.** Reaction free energy (in eV vs RHE) of elementary step at U_RHE_=0V and onset potential (in V vs RHE) for ORR on different on O-reconstituted Cu SACs.

|  | 𝛥𝐺_1_ | 𝛥𝐺_2_ | 𝛥𝐺_3_ | 𝛥𝐺_4_ | U_onset_ |
| --- | --- | --- | --- | --- | --- |
| *1pd_N-3pd_C-O* | -0.35 | -3.06 | -0.17 | -1.33 | 0.17 |
| *2pd_N-2pd_C-side1-O* | -0.84 | -2.00 | -0.91 | -1.17 | 0.84 |
| *2pr_N-2pd_C-O* | -0.27 | -2.55 | -0.39 | -1.71 | 0.27 |
| *3pd_N-1pd_C-O* | -0.65 | -0.94 | -2.40 | -0.94 | 0.65 |

**Reference**

1. G. Kresse and J. Furthmüller, *Comput. Mater. Sci.*, 1996, 6, 15-50.

2. G. Kresse and J. Furthmüller, *Phys. Rev. B*, 1996, 54, 11169.

3. P. Steneteg, I. Abrikosov, V. Weber et al., *Phys. Rev. B*, 2010, 82, 075110.

4. G. Pacchioni, L. Giordano and M. Baistrocchi, *Phys. Rev. Lett*, 2005, 94, 226104.

5. K. Mathew, R. Sundararaman, K. Letchworth-Weaver, T. Arias and R. G. Hennig, *J. Chem Phys.*, 2014, 140, 084106.

6. H. Xu, D. Cheng, D. Cao, X. Zeng, *Nature Catal.* 2018, 1, 339-348.

7. L. Yang, D. Cheng, H. Xu, X. Zeng, X. Wan, J. Shui, and D. Cao, *PNAS,* 2018, 115, 6626-6631.
